# Feline‐Inspired Robot Enabled by Combustion‐Driven Actuators for Agile Motion and High‐Payload Obstacle Traversal

**DOI:** 10.1002/advs.202519885

**Published:** 2025-12-26

**Authors:** Hongkuan Ma, Yang Yang, Zhiguo He, Hongliang Ren, Pengcheng Jiao

**Affiliations:** ^1^ State Key Laboratory of Ocean Sensing & Ocean College Zhejiang University Zhoushan Zhejiang China; ^2^ Hainan Institute Zhejiang University Sanya Hainan China; ^3^ Engineering Research Center of Oceanic Sensing Technology and Equipment Ministry of Education Zhejiang University China; ^4^ Department of Electronic Engineering Faculty of Engineering The Chinese University of Hong Kong Hong Kong China

**Keywords:** bionic robot, combustion‐driven soft actuator, high‐force environmental interaction, versatile locomotion

## Abstract

In nature, many animals and skilled humans perform rapid, high‐force maneuvers such as strikes, leaps, or escape responses, through instantaneous energy release. Replicating this synergy of speed, power, and precision in soft robots has remained an unsolved challenge. Here we introduce a combustion‐driven soft actuator with an embedded backbone that, using a transient driving method, delivers millisecond‐scale response, force outputs up to 70 times its self‐weight, and control precision within 5%. This capability supports both static tasks (e.g., precision throwing), and dynamic actions (e.g., intercepting moving targets). Leveraging this actuator, we developed a feline‐inspired robot that accelerates to eight body lengths per second and transitions to flight within 0.1 s, creating a multi‐modal “Jump‐and‐Fly Catbot” (JFC) capable of jumping, flying, and hovering. JFC navigates unstructured terrains and demonstrates robust escape capabilities, including freeing itself from bed entrapment, avoiding obstructing branches, and evading net capture, with response within 0.5 s, that surpass current soft robotic systems. These results establish a new paradigm for soft robots, integrating high‐force interaction, precision control, and versatile locomotion for robust operation in complex environments.

## Graphical abstract

Inspired by nature's competitive maneuvers, this study introduces a combustion‐driven soft actuator that powers a multi‐modal “Jump‐and‐Fly Catbot” (JFC). With millisecond response, high‐force output (over 70 times its weight) and precise control (error within 5%), the robot can jump, fly, hover, and escape from challenging environments, achieving agility and precision beyond existing soft robotic systems.

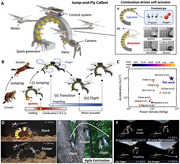

## 1 Introduction

Endowing soft robots with instantaneous high‐force generation requires overcoming the innate limitations of flexible materials—particularly their low energy density actuations [1]. Achieving such rapid energy release addresses a fundamental challenge: replicating and advancing beyond biological instantaneous motions [2, 3, 4], such as jumping, predation, and escape. Current approaches employ two primary strategies: chemical reaction driven high‐energy release [5, 6, 7] or mechanically tuned energy storage‐release cycles [8, 9]. Through sudden energy‐release actuation, existing soft robots can now execute these demanding tasks [10, 11].

However, beyond mobility, it is challenging to balance delicate environmental interaction with high‐force actuation and precise control [12]. Such rapid, powerful environmental interactions are ubiquitous in nature, yet remain difficult for soft robots to achieve. For example, creatures utilize projectile ejection motions for reproduction, defense, and predation [13]. Such instantaneous high‐force interactions also enable creatures to escape external confinement: shrimps (e.g., odontodactylus scyllarus) generate over 1500 N (∼2500 × body weight) strike force within 20 ms to fracture corals, breaking free from physical entrapment [14]. This limitation stems from insufficient interaction time during high‐speed robotic motions with static/dynamic objects, making accurate control of powerful soft robots during environmental interaction the core problem [15].

Addressing this challenge requires a systematic study from actuation principles to application strategies. Regarding the fundamental level, a powerful soft actuator with precise controllability is essential, which the current studies mainly focus on. Combustion‐driven actuation using the transient driving method (TDM) offers a proven solution: igniting combustible premixed gas rapidly converts chemical energy into instantaneous high pressure, driving large deformations in soft materials [16, 17, 18]. Compared with the mechanical energy‐release systems (e.g., nylons, springs, and magnets), combustion achieves higher power density (277.2 kW/kg vs. 0.73 kW/kg, combustion vs. nylons) [19], more powerful actuation forces (22 × body weights vs. 6 × body weights, combustion vs. springs) [20], and faster cycling frequencies (100 Hz vs. 60 Hz, combustion vs. magnets) [8], which is critical for high‐power dynamic interactions. At the functionality level, achieving effective object interaction within microsecond‐scale temporal windows presents a critical challenge [21, 22]. The inherently explosive actuation velocity poses difficulties for precise control during interactions with both static and dynamic objects. For example, precise targeting of static objects (e.g., projectile throwing) and accurate interception of moving targets (e.g., catching an object at 3 m/s within 40 ms through a Venus‐flytrap‐inspired soft gripper [23], though with limited high‐output force precision for striking interference objects), while simultaneously enabling rapid shape recovery. At the application level, modular integration of these high‐force interaction systems into existing robotic platforms unlocks new potentials. This approach enables multimodal locomotion capabilities while significantly enhancing environmental interaction, such as evading terrain constraints, escaping external threats, and addressing critical needs in unstructured environments [24, 25, 26]. Previous researchers have integrated hopping and flying capabilities (e.g., flapping‐wing and passive spring‐based leg [27], quadrotor and passive telescopic leg [28]), enhancing the multi‐terrain adaptability of robots, yet achieving high output force with precise control to escape from environmental constraints remains a challenges.

This study systematically addresses these challenges through a combustion‐driven soft actuator incorporating an embedded backbone structure. The actuator achieves a rapid response within 3–5 ms, force output up to 70 times its body weight, and control accuracy within 5%, enabling precise interaction with environmental objects. Combustion‐backbone coupling induces asymmetric stress propagation that produces powerful yet controllable bending motions. Building on this principle, we developed a lightweight (∼95 g) prototype that demonstrates both static object ejection and dynamic object interception of moving targets, validated through experiments and simulations. Extending this design, we created the Jumping‐and‐Flying Catbot (JFC), a multimodal platform that integrates terrestrial and aerial capabilities. This hybrid design enables JFC to overcome the limitations of conventional drones and jumping‐only robots by synergizing high‐force instantaneous jumping with sustained and controllable flight. Experimental tests confirm JFC's ability to overcome environmental constraints, such as bed or branch entrapment, through combustion‐powered jumps. Its rapid 0.1 s jump‐to‐flight transitions enable effective evasion in complex environments, outperforming conventional UAVs and existing jumping soft robots.

## 2 Results

### 2.1 Design Principle and Actuation Mechanism

**FIGURE 1:**
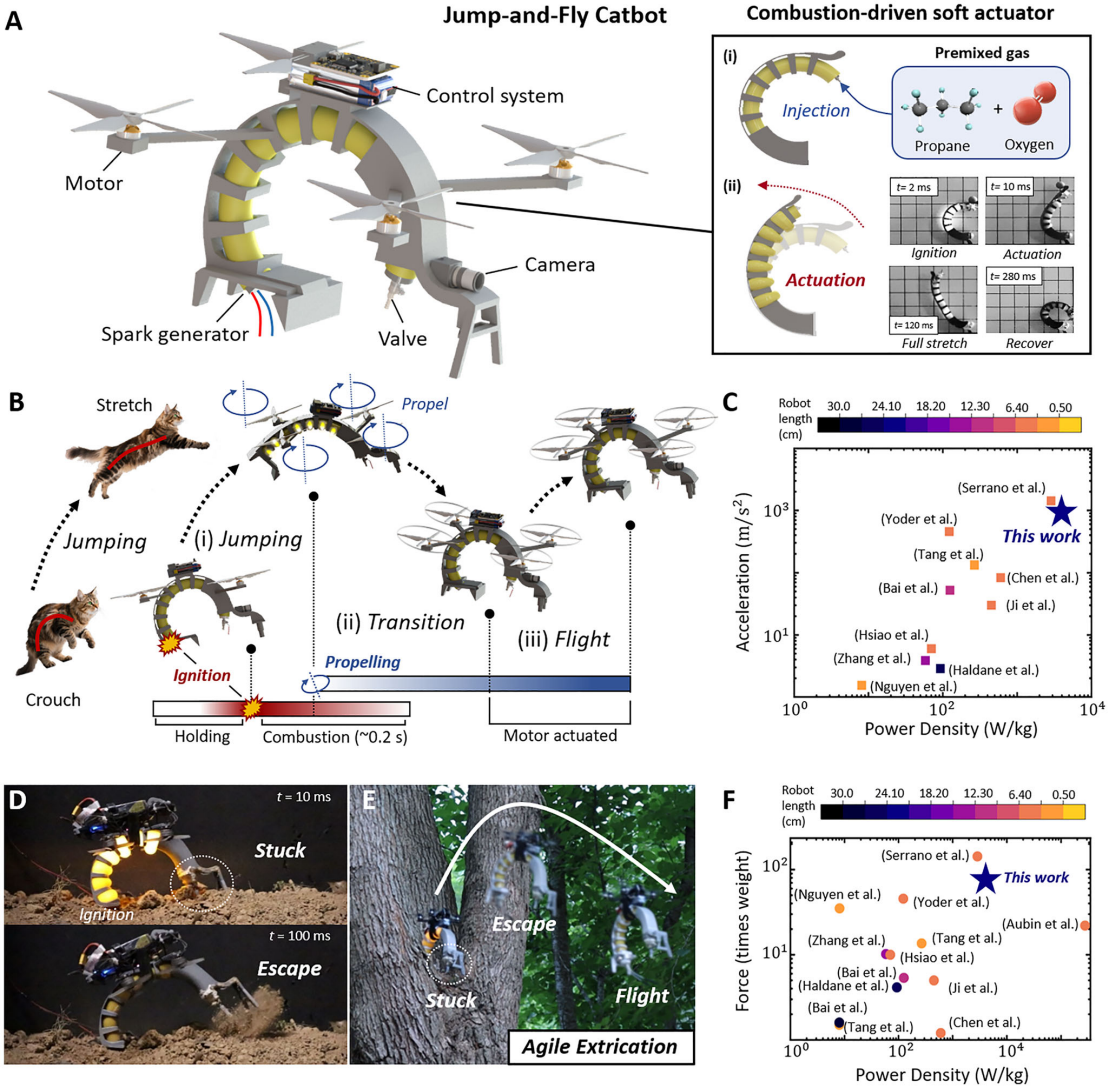
Illustrative demonstration of the Jump‐and‐Fly Catbot (JFC) and combustion‐driven soft actuator. (A) Design principles of the JFC and combustion‐driven soft actuator. (B) The driving mechanism of the JFC: jumping, transition, and flight process. The jumping actuation of the JFC is inspired by the jump motion of felines. (C, F) Jumping performance comparisons with existing soft robots [2, 5, 8, 9, 10, 11, 27, 28, 31, 32, 33, 34, 35, 36, 37], showing JFC's power density (3730 W/kg) and output force (65.36 N, 70 × self weights). (D) Rapid escaping from environmental constraints (e.g., sand and stones) when trapped in unstructured terrains. (E) Agile extrication from entangled branches demonstration.

The combustion‐driven soft actuator (total mass: 95 g) comprises a backbone structure and an inner balloon, with dimensions and structural details illustrated in Figure 1A and Figure S1A. A valve mounted on the balloon head enables premixed gas injection. The backbone features six semicircular ribs (diameter: 25 mm) tilted 80° from the central axis to constrain radial balloon expansion. Additionally, a base provides a fixed constraint to the actuator, while the backbone wall connects these components. This asymmetric design converts the balloon's isotropic expansion into anisotropic deformation of the actuator within 0.01 s, producing rapid bending. The balloon length (250 mm) and outer diameter (24 mm) are matched to the rib geometry, ensuring adequate premixed gas volume.

The combustion‐driven actuator mechanism is illustrated in Figure S1B. The entire process occurs within 0.3 s and comprises four stages: ignition, actuation, stretching, and recovery. Premixed oxygen‐propane gas (ratios 3:1, 4:1, or 5:1) is first injected into the balloon [7], which initially has limited backbone contact. Upon ignition, the balloon expands radially and axially within 0.01 s. As combustion completes, the balloon presses against the ribs, and asymmetric stress distribution drives rapid bending. After reaching maximum stretch, the actuator passively recovers to its original shape.

We further designed a Jumping‐and‐Flying Catbot (JFC), a multimodal platform based on the combustion‐driven soft actuator with electric propellers to integrate terrestrial and aerial locomotion. The control system, including the flight controller, battery, and receiver, is mounted on the upper exoskeleton, as demonstrated in Figure 1A. JFC measures 20 cm in length with a 15 cm motor span. Its locomotion mechanism (Figure 1B) draws inspiration from feline biomechanics: the backbone is pre‐curled to mimic a cat arching its spine to store elastic energy before a jump [29, 30]. Upon combustion, the balloon rapidly expands, asymmetric backbone deformation generates a leap, after which the propellers engage to stabilize flight. Comparative analyses (Figure 1C,F) highlight rapid actuation and high thrust, achieving a peak power density of 3730 W/kg and forces up to 65.36 N. Additional experiments (Figure 1D,E) reveal the JFC's ability to extricate itself and navigate across unstructured and cluttered terrains, tasks difficult for propeller‐driven locomotion alone.

### 2.2 Combustion‐Driven Actuation

**FIGURE 2:**
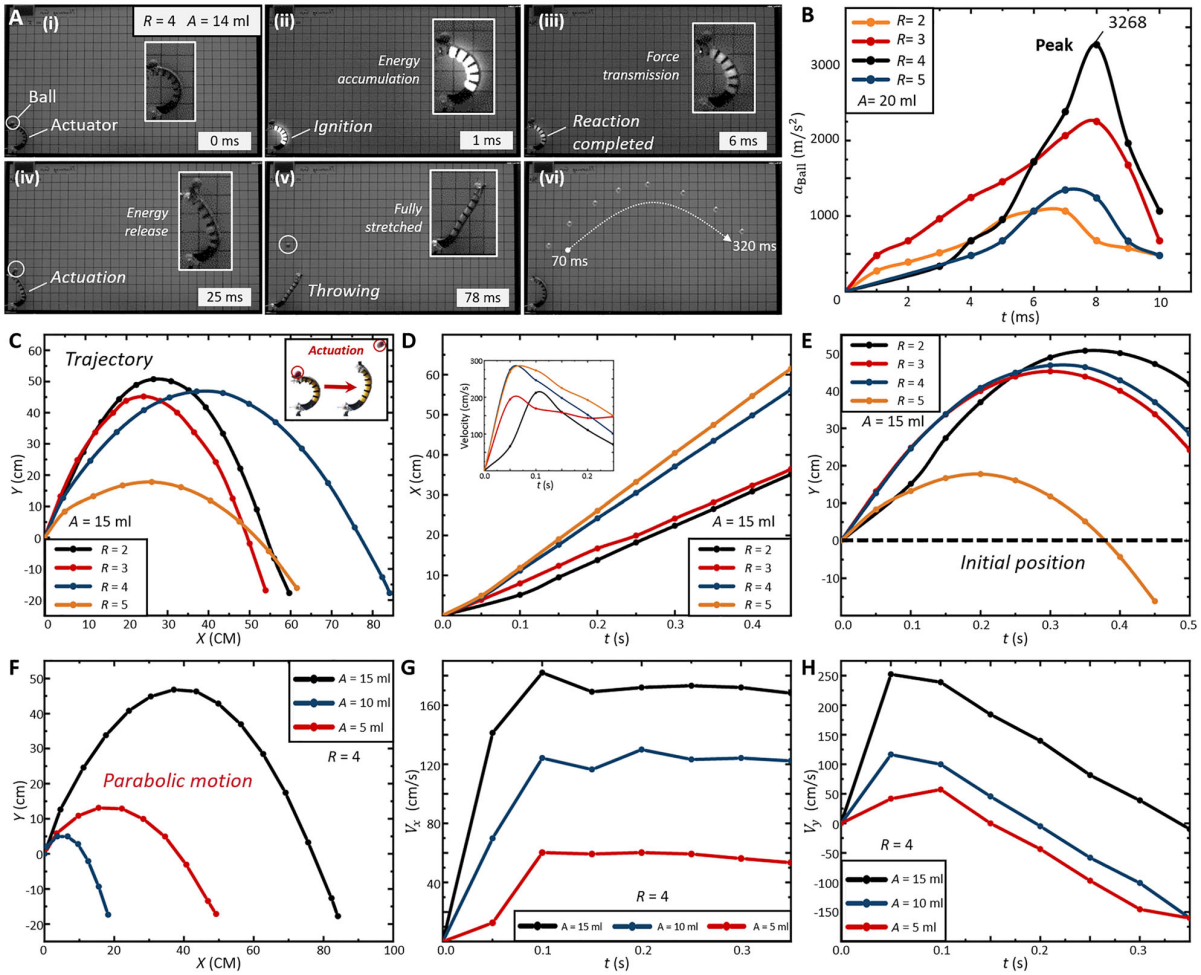
Actuation experimental setup and results. (A) The photographs of the combustion‐driven soft actuator's actuation experiment. (B) The maximum acceleration of the object when the ratio of premixed gas (*R*) is 2, 3, 4, and 5, and the gas amount (*A*) is 20 mL. The maximum acceleration *a*_Ball_ is up to 3268 m/s^2^, achieving a payload of 65.36 N, which is up to 70 times its self‐weight. (C) The trajectory of the object, when the *A* is 15 mL, exhibits a parabolic shape. (D) Horizontal motion of the object. (E) Vertical motion of the object. (F) The trajectory of the object when *R* is 4 with different gas amounts. (G) Horizontal velocity of the object. (H) Vertical velocity of the object.

To investigate the influence of key parameters on actuation performance, we conducted experiments varying the premixed gas ratio *R* and gas amount *A*. Figure 2A shows the actuation sequence for a gas ratio *R* = 4 and a volume *A* = 14 mL (see Figure S2 and Movie S1). At 0.001 s, combustion initiates inside the balloon, causing a rapid pressure rise and simultaneous radial and axial expansions. During the deformation process, the asymmetric backbone structure constrains balloon expansion, generating uneven bending stress distribution that actuates a rapid throwing motion. The actuator outputs thrust to a 20 mm diameter, 20 g ball, producing rapid acceleration within 0.01 s. It should be noted that byproducts (e.g., soot, CO_X_ compounds, NO_X_ compounds) are produced during the reaction process [5], which leads to insufficient reactions. Therefore, it is necessary to investigate the effect of different ratios (*R*) of premixed gas on actuation performances (see Table 1, and the reaction mechanism is shown in Text S1). The object acceleration under different combustible gas ratios has been demonstrated in Figure 2B, and the *A* is 20 mL. The instantaneous acceleration of the object reaches up to 3268 m/s^2^, with an actuating force of 65.36 N, and the payload is up to 70 × body weights. Optimal actuation occurs at *R* = 4 under the same gas volume (see Figure 2C–E).

**TABLE 1:** Case table of actuating tests.

Case	1	2	3	4	5	6	7	8	9	10	11	12
Amount (ml)	5	10	15	5	10	15	5	10	15	5	10	15
Ratio	2:1	2:1	2:1	3:1	3:1	3:1	4:1	4:1	4:1	5:1	5:1	5:1

Furthermore, previous studies suggest that actuation performance scales positively with premixed gas amount [7] (see setup in Table 1). At a fixed gas ratio, object acceleration increases monotonically with gas volume. Figure 2F–H presents the ball trajectories under constant ratio and increasing gas amount, which demonstrates that the premixed gas amount is a critical determinant of actuation. We further quantified combustion dynamics through internal pressure measurements, establishing empirical relationships linking pressure, premixed gas ratio, and gas amount with errors below 5% (Figure S27, and Text S9).

The combustion‐driven actuator generates a peak force of 65.36 N (∼70 × body weight) with a millisecond‐scale response—performance unattainable by electrohydraulic actuators, which typically operate at kilovolt levels yet still produce limited force and displacement. [38, 39] Moreover, the power density of our combustion‐driven soft actuator reaches 3730 W/kg, significantly surpassing that of typical pneumatic actuators (≤300 W/kg) [1, 40, 41], thereby enabling dynamic tasks such as precise interception of moving targets. While low‐voltage liquid metal actuators represent an alternative approach, their dependence on electrolytes leads to relatively low stress output (on the kilopascal scale) and lower power density compared to our actuator, which operates at megapascal‐level equivalent stress [42].

### 2.3 Numerical Results

**FIGURE 3:**
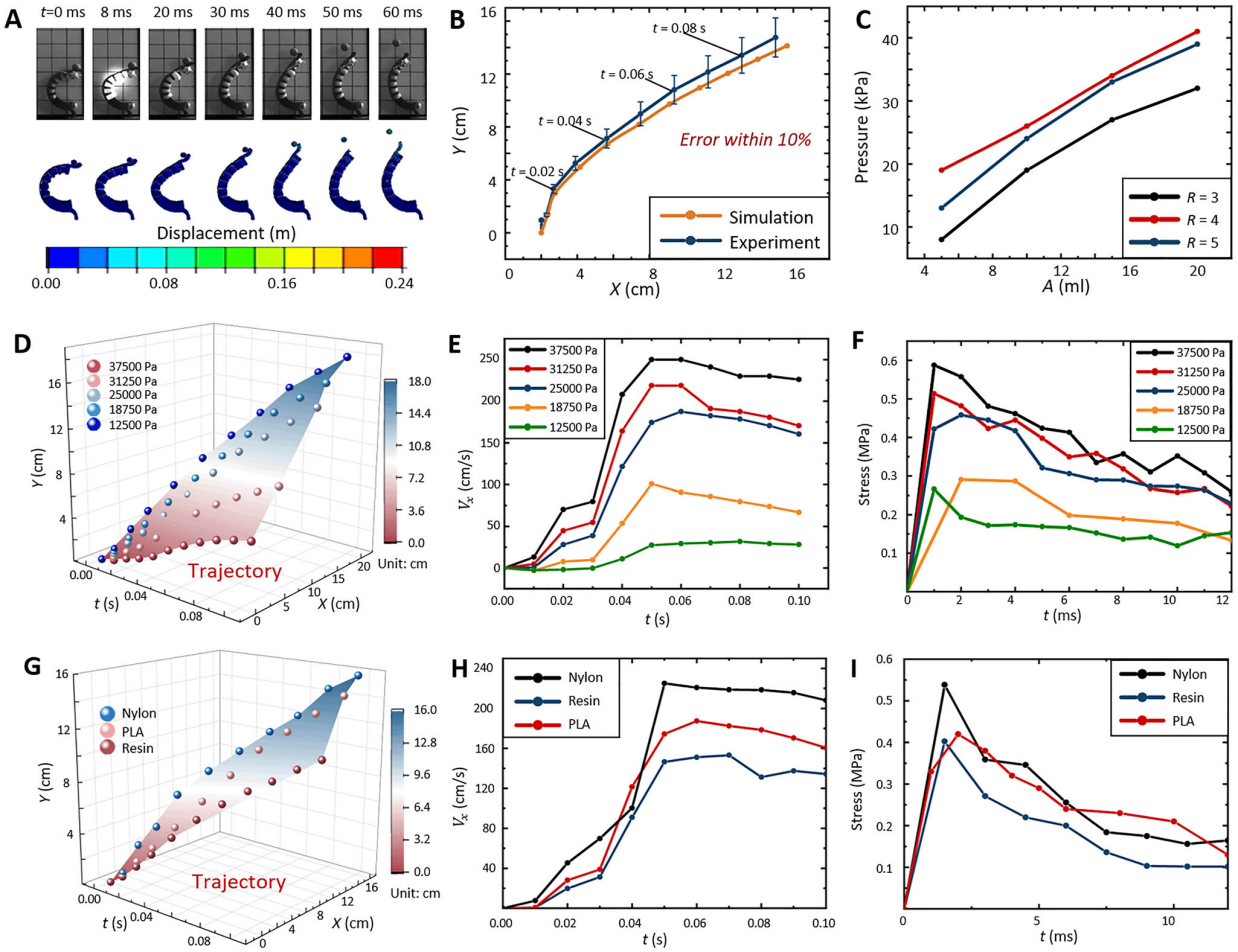
Numerical setup and results. (A, B) Comparisons between numerical and experimental results (trajectory error within 10%). (C) Internal pressure evolution of the combustion‐driven soft actuator during the actuation process. (D–F) Thrown trajectory, velocity, and the stress in the contact region at varied combustion pressures. (G–I) Thrown trajectory, velocity, and the stress in the contact region for different backbone materials.

The numerical results were validated experimentally and showed favorable agreement with the observed trajectories of thrown‐ball motion (Figure 3A,B). A barometer (range: 0–200 kPa) measured internal balloon pressure at a gas ratio of *R* = 4 under varying gas amounts (Figure 3C). For a volume of 20 mL, the internal pressure reached ∼41 kPa, with detonation waves propagating at 2500 m/s (see Text S2 for the relationship between detonation velocity and pressure) [43]. This corresponds to a transmission time of 0.04 ms within the balloon. Given that this duration is negligible, a uniform combustion pressure of 41 kPa was therefore applied in the numerical model. Ball trajectory validation within 0.1 s confirmed the model's reliability with trajectory errors below 10%.

The motion trajectories of the object under different initial combustion pressures are displayed in Figure 3D,E. To investigate the actuation mechanism, we analyzed the time‐dependent stress in the contact region, as shown in Figure 3F. The stress peaks within 2 ms after ignition, followed by a gradual trend. As the combustion pressure increases, the displacement and the maximum velocity of the object also increase (as detailed in Table S1) due to the increased stress on the backbone. When the combustion pressure reaches 37.5 kPa, the maximum velocity of the object reaches a maximum velocity of 3.4 m/s. The stress in the contact region is up to 0.6 MPa, and the detailed object trajectory is shown in Figure S3.

To investigate material effects on actuation performance, simulations were performed with nylon, resin, and PLA backbones (see Figure 3G–I), and detailed trajectories are shown in Table S2 and Figure S4. The nylon‐actuator exhibited the highest acceleration and velocity at the same combustion pressure (26.25 kPa). This phenomenon can be attributed to the low elasticity modulus of nylon, which increases backbone flexibility. Furthermore, under identical conditions, the nylon‐actuator provides stronger interaction forces with the object (contact stress ∼0.53 MPa) than Resin (∼0.41 MPa) or PLA (∼0.43 MPa). These results identify nylon as the optimal backbone material, offering a significant improvement in actuation performance.

### 2.4 Throwing and Hitting

We conducted two demonstrations to validate the actuator's capacity for rapid, stable, and controllable bending motions. Such powerful object interactions confirm its viability as the core of the feline‐inspired robot. In the three‐point‐shot task, the actuator demonstrated stability, precise controllability, and fast response. In the baseball‐home run task, it generated high payload forces while accurately intercepting moving targets. Both demonstrations highlight capabilities, i.e. instantaneous force output, precise control, and dynamic interaction, that remain challenging for previously reported bionic soft robots due to material compliance limitations in force, speed, and controllability [32, 44]. These two demonstrations thus effectively validate the actuator's ability to deliver powerful, precise, and stable object interactions beyond the reach of existing soft robotic system.

**FIGURE 4:**
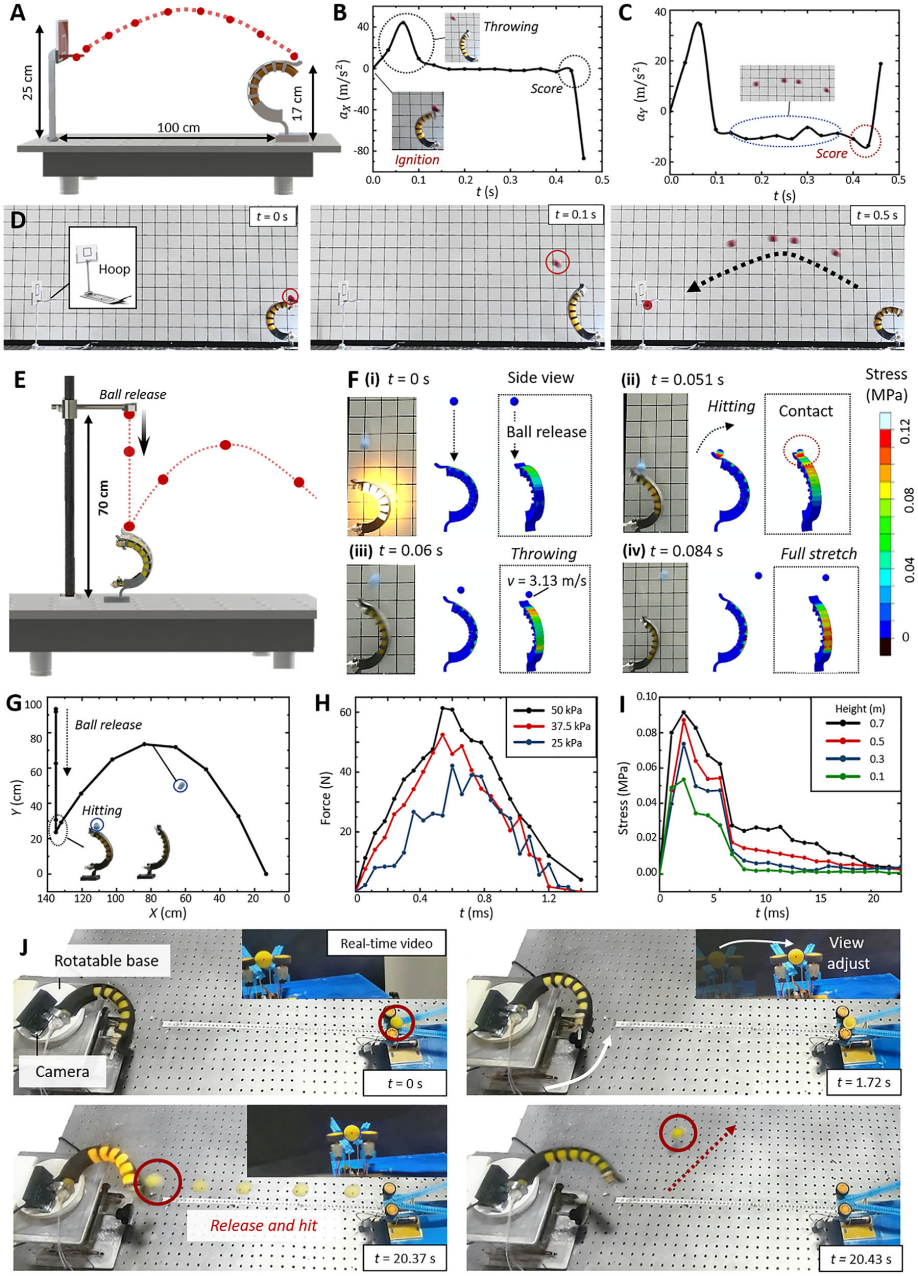
High‐force object interaction capability validation by the three‐point shot and baseball home run demonstrations. (A) Experimental setup for the three‐point‐shot demonstration. (B) Relationship between acceleration and time in the horizontal direction. (C) Relationship between acceleration and time in the vertical direction. (D) Photographs of the three‐point shot demonstration. (E) Experimental setup of the baseball home run. (F)Simulation of the baseball home run at *t* = 0, 0.051, 0.06, and 0.084 s. (G) Trajectory of the baseball motion. (H) Resultant force of the baseball at the contact stage. When the pressure is 50 kPa, the force reaches up to 60 N. (I) The stress on the contact region. (J) Horizontal sensing‐actuation demonstration: A miniature camera is installed on the actuator, which can accurately identify ball positions. When the ball diameter in the camera increases to the threshold, the actuator is activated and accurately hits the ball.

The three‐point‐shot demonstration exhibits the combustion‐driven soft actuator's advances over existing designs through balloon‐backbone integration, significantly enhancing controllability and stability for high‐force object manipulation. As shown in Figure 4A, the actuator is positioned 100 cm from the basket with a release height of 17 cm. Guided by prior results, 15 mL of premixed gas (*R* = 4) was injected. The resulting motion produced a parabolic trajectory of the ball (Figure 4D; Movie S2). Horizontal velocity peaks at 2.5 m/s within 0.1 s (Figure 4B,C), while vertical velocity reaches 1.8 m/s before gravitational deceleration. The ball attains maximum height at 0.25 s and contacts the rim at 0.42 s.

To validate moving‐target interception, we designed the baseball‐home run demonstration, in which the actuator intercepted an incoming ball. Implementing such capabilities has been a long‐standing change for soft robots due to material compliance, limited power density, and difficulty of millisecond‐scale actuation [23, 45, 46]. Here, integration of an optical sensing‐response system with the combustion‐driven actuator enabled rapid perception and instantaneous force generation, delivering high payload forces with precise timing. This approach demonstrates a viable pathway to overcome fundamental limitations in dynamic, high‐force object interactions.

Figure 4E illustrates the baseball home run demonstration setup, with the baseball initially positioned 70 cm above the base. The trajectory of the baseball is presented in Figure 4G, with velocity and acceleration profiles detailed in Figures S5–S7 and Text S3. From 0 to 0.26 s, the ball is in free‐fall motion, reaching a peak downward velocity of 3.13 m/s. At 0.26 s, as the baseball approaches the actuator, the combustion is triggered (*R* = 5, *A* = 20 mL), enabling precise interception (see Movie S3). To further investigate the mechanism of interaction, we conducted simulations (Figure 4F). Upon ignition, backbone stress rapidly increases, and then the actuator hits the ball at 0.051s. The contact, lasting 1.5 ms, generates a peak resultant force at 0.5 ms (Figure 4H). At 50 kPa combustion pressure, the maximum resultant force of the baseball reaches up to 60 N. For a baseball drops from 0.7 m, contact stress reaches 92 kPa, providing powerful output to rapidly redirect the ball's motion (Figure 4I; Figure S8).

To validate precise controllability and optical sensing‐actuation integration, we conducted experiments combining optical recognition with targeted actuation. In the horizontal demonstration (Figure 4J), the actuator equipped with a micro‐camera was mounted on a rotating platform, with a ball thrower positioned 60 cm away (Figure S9B). At *t* = 0 s, the camera detected off‐center ball positioning, triggering platform rotation, until the actuator targeted the ball. Following ball throwing toward the actuator, combustion was activated at *t* = 20.37 s when the ball diameter reached the recognition threshold, resulting in precise hitting (Movie S4). The vertical demonstration is detailed in Text S4. These results confirm the potential of the integrated optical sensing‐actuation system for bionic soft robots requiring precise control, millisecond‐scale response, and high‐force interaction.

### 2.5 Motion Performance of the Jump‐and‐Fly Catbot

Designing soft robots that can perform complex biomimetic motions for agile extrication and navigation across uneven terrains, obstacles, and chasms remains a significant challenge. To address this, we developed the Jump‐and‐Fly Catbot (JFC), which executes motions such as lifting its front feet, stomping its rear feet, and contracting during the jump phase to achieve agile extrication and rapid evasion.

**FIGURE 5:**
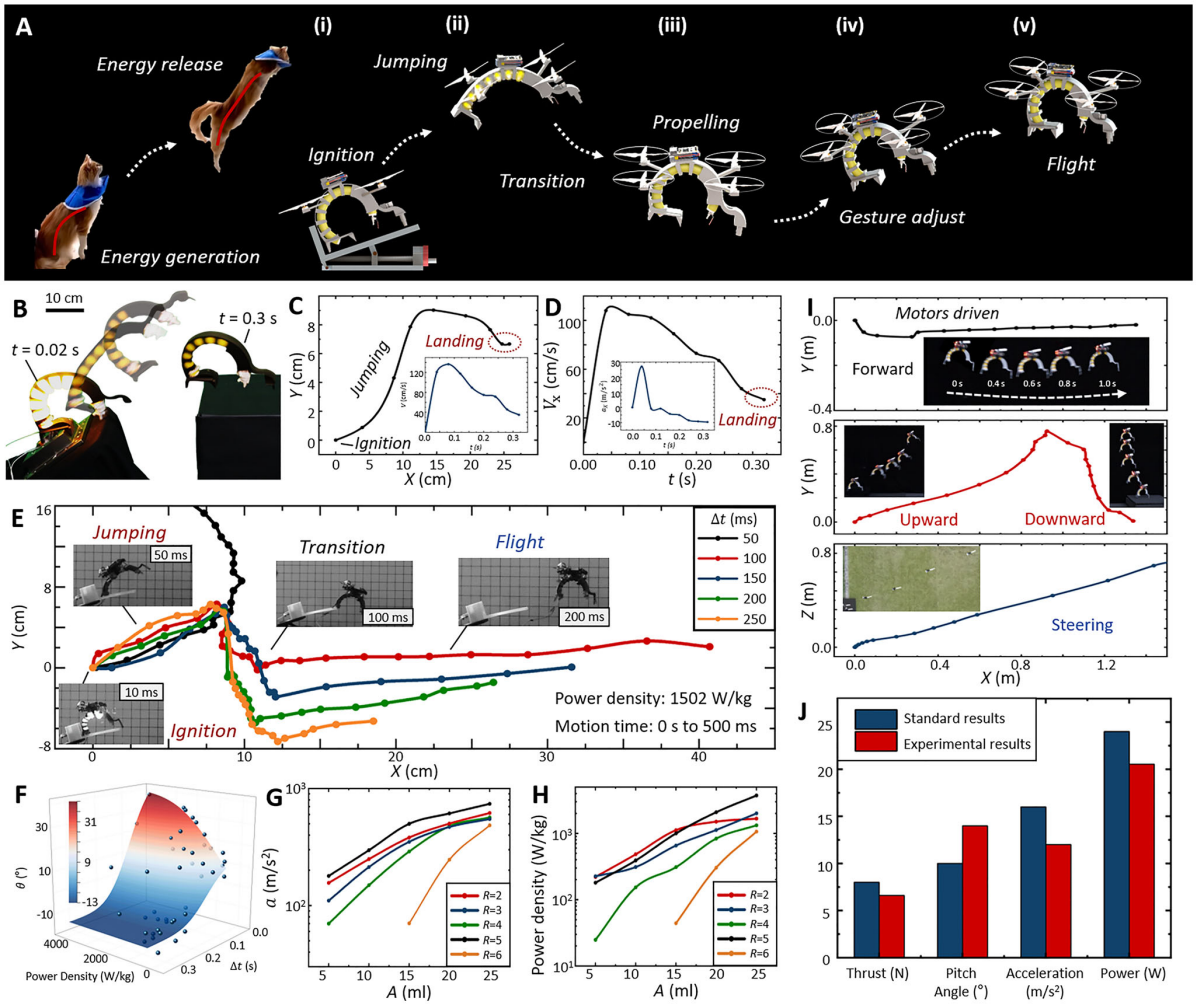
Jump‐flight locomotion performance. (A) Motion mechanism of the JFC. (B) Jumping process. (C, D) Trajectory and velocity of the jumping process. (E) The motion trajectory of the JFC jumping and flying. The interval time (Δ*t*) between jumping and flying is 50, 100, 150, 200, and 250 ms. The power density is 1502 W/kg, and the entire motion time is 500 ms. (F) The elevation angle at different power densities and interval times. The maximum elevation angle is 30°, when the power density is 3730 W/kg. (G, H) The jumping acceleration and power densities. When *R* = 5 and *A* = 25 mL, the JFC achieves a peak power density of 3730 W/kg and generates instantaneous leaping acceleration up to 800 m/s^2^. (I) The flight capability of the JFC. (J) The comparative analysis of the flying motion between the standard results and experimental results.

The JFC integrates a combustion‐driven soft actuator and four electric blades, forming a multimodal platform that combines terrestrial and aerial locomotion. Its motion integrates a combustion‐driven actuation for rapid hopping, followed by motor activation for flight (Figure 5A). Notably, during rapid jumping, the robot achieves a velocity of eight body lengths per second (BLPS) with instantaneous acceleration up to 800 m/s^2^ (Movie S5). Under optimal premixed gas conditions (*R* = 5, *A* = 25 mL), the JFC attains a peak power density of 3730 W/kg (Text S5 and Movie S6). Mimicking feline biomechanics, the JFC employs a curled‐spine configuration to concentrate thrust (Figure 5B–D).

To investigate the motion transition between jumping and flight, we systematically modulated the temporal intervals (Δ*t*) between combustion actuation and propeller activation while tracking trajectories at a power density of 1502 W/kg (Figure 5E). Analysis reveals that transitions with Δ*t* ≤ 0.05 s initiate immediate upward flight post‐jump, whereas intervals exceeding 0.1 s led to a ballistic descent process before ascent. Figure 5F characterizes the JFC's pitch angle variation across parametric power densities and transition intervals, with wing elevation angles (θ_A_: forewing, θ_B_: tailwing) quantified in Figure S13. This reveals an inverse correlation where reduced transition intervals yield increased pitch angles at constant power density. Figure 5G,H illustrates the JFC's acceleration and power density under varying gas amounts and premixed gas ratios. Comparative analysis (Figure 5I,J; Text S6 and Movie S7) further demonstrated the JFC's aerial capabilities, confirming stable flight, controlled hovering, and effective navigation across unstructured terrain.

### 2.6 Rapid Escape from Environmental Constraints

Lightweight soft robots can leap over obstacles [5, 31, 47], yet they typically lack advanced motion capabilities, such as rapid extrication, evasion, and hovering. Comparative power‐density analysis [5, 19, 48] shows that the combustion‐driven actuation (> 2000 W/kg) substantially outperforms nylon‐based (∼700 W/kg) and dielectric elastomer (∼600 W/kg) approaches, providing sufficient energy for a powerful jump in unstructured and cluttered environments.

**FIGURE 6:**
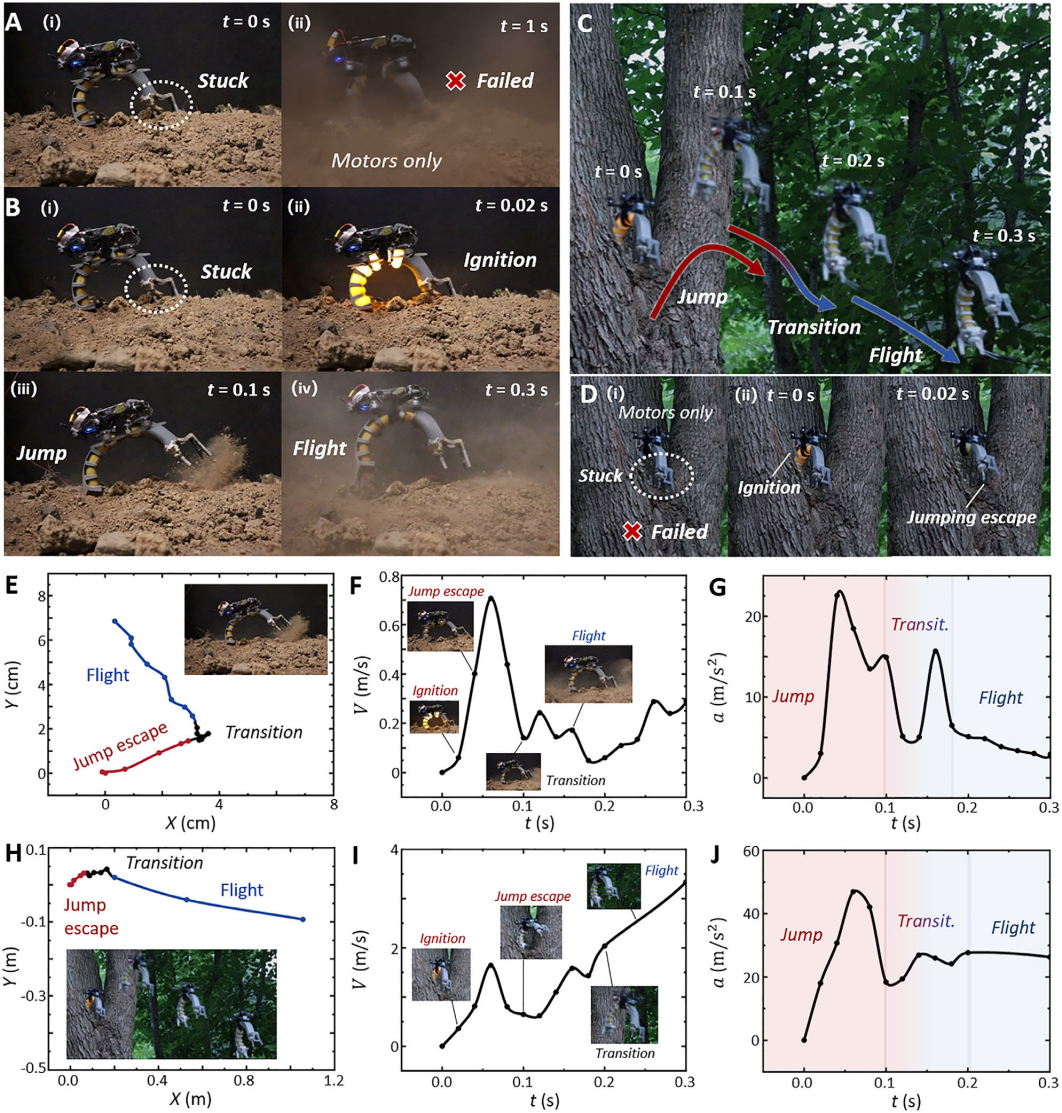
JFC escaping environmental constraints. (A) JFC stuck in sand; lift from the electric blades alone is insufficient for escape. (B) JFC jumping from unstructured terrain (sand). Combustion actuation at 0.02 s provides high power density (3730 W/kg), enabling the JFC to jump free by 0.1 s. (C) JFC performing agile extrication from entangled branches. (D) In contrast, a conventional UAV stuck in branches uses the combustion‐driven method for rapid disentanglement, enabling continued flight. (E–G) Track, resultant velocity, and resultant acceleration during sand escape. Maximum velocity reaches 0.7 m/s within 0.1 s, enabling rapid escape. (H–J) Corresponding track, resultant velocity, and resultant acceleration during rapid leaping from tree branches.

The JFC demonstrates this capability by escaping from sandy terrain constraints and transitioning to flight (Figure 6B). When trapped in complex terrain, rotor thrust alone proved insufficient for takeoff (Figure 6A). Combustion actuation, however, generated the additional thrust need for extrication, enabling rapid jumps from sand, followed by sustained flight (trajectory in Figure 6E). During jumping, the JFC reached 0.7 m/s velocity and 22 m/s^2^ acceleration, as shown in Figure 6F,G. Upon completion of the jump, the motor activated with 15 m/s^2^ acceleration (Movie S8).

The combustion‐driven method enables rapid escape from branch entanglement (Figure 6C; Movie S9). Conventional unmanned autonomous vehicles (UAVs) often rely on oscillation or external recovery [49, 50], yet remain inefficient and prone to failure. Operating solely on electric power, the UAV could not free itself from the entanglement of branches (Figure 6D). By contrast, combustion‐driven thrust allowed the JFC to break free within 0.1 s and transition into stable flight. To mitigate entanglement risks in arboreal environments, we implemented an untethered actuation system (Figure S19). The corresponding kinematic trajectories and performance during rapid extrication are illustrated in Figure 6H–J.

### 2.7 Rapid Evasions

**FIGURE 7:**
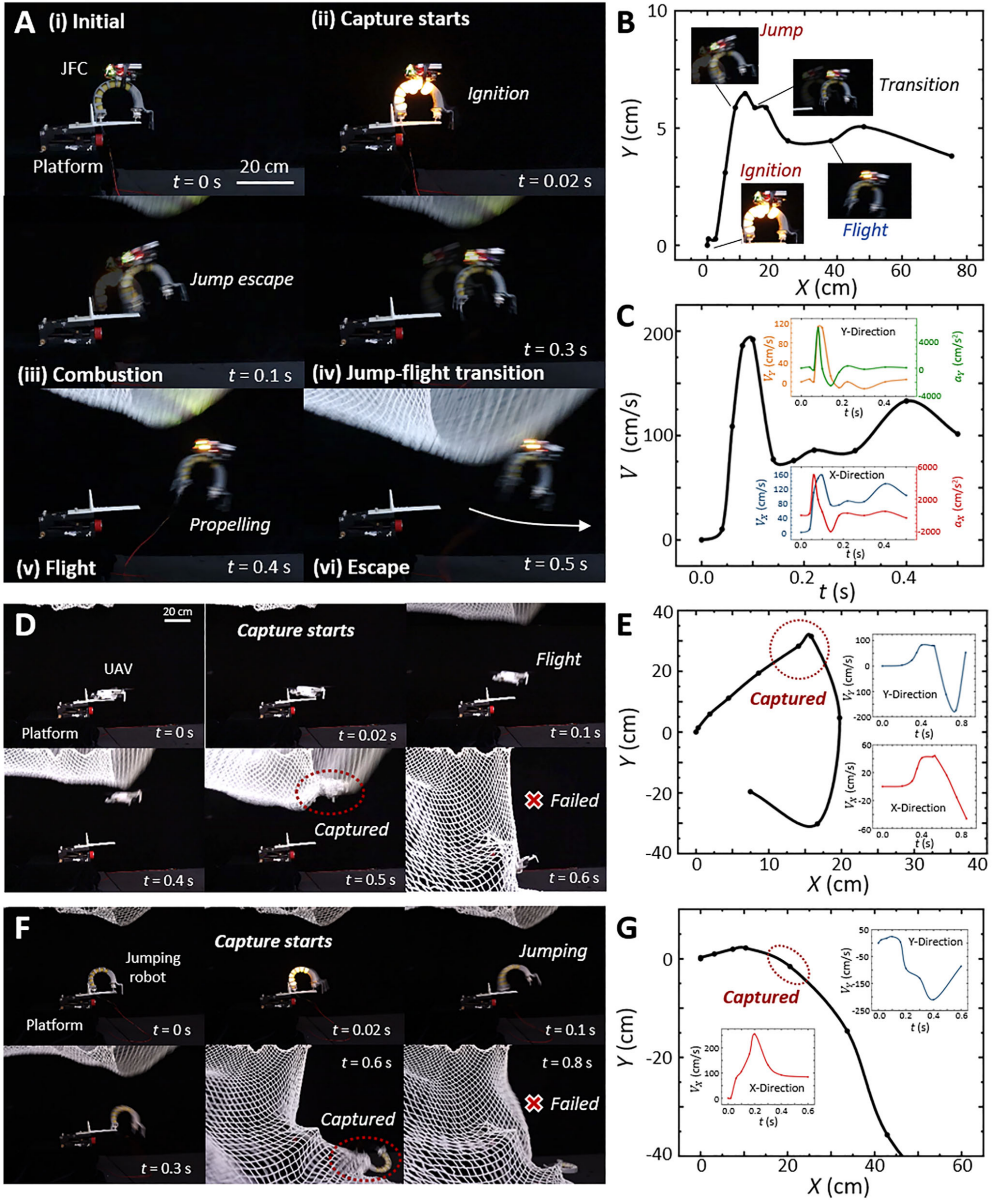
JFC rapid evasion demonstration. (A) Demonstration of the JFC rapid evasion catching net. (B) The trajectory of the JFC rapid evasion catching net. (C) The kinematics of JFC rapid evasion (e.g., velocity and acceleration). (D, E) The conventional UAV failed to evade the net due to the limited transient power density and response time. (F, G) The jumping‐only robot failed to escape from the net due to inadequate sustained flight capability. Demonstrations highlight that when confronted with a descending net, only the JFC can successfully evade, which validates the necessity of integrating jumping and flying capabilities.

To validate the JFC's rapid evasion and agile mobility under external interference, we conducted a comparative experiment with the JFC, a conventional UAV, and a jumping robot, each challenged by descending capture nets (Movie S10). Upon net initiation at *t* = 0.02 s, the JFC triggers combustion actuation and transitions to flight by *t* = 0.3 s, successfully evading the capture net (Figure 7A). Its kinematic trajectory (Figure 7B,C) shows a peak leaping velocity of 2 m/s. By contrast, the conventional UAV, limited by power density and slower time, reaches only 0.89 m/s and failed to escape the net (Figure 7D,E). The jumping robot displays fast actuation and leaping but, lacking sustained aerial locomotion, is also captured (Figure 7F,G). This comparison highlights the necessity of integrated combustion‐electric actuation, which enables the JFC to achieve rapid evasion beyond the capacity of conventional UAVs and jumping soft robots in constrained environments.

## 3 Conclusion

This study developed a novel combustion‐driven soft actuator integrated with an embedded backbone structure that delivers ultrafast response (3–5 ms), high output force (∼70× self‐weight), and precise controllability (≤5% error), enabling strong and versatile environment interaction. We systematically examined how premixed gas parameters affect actuation performance and developed a numerical model, validated by experiments, that clarified the actuation mechanism and backbone material effects, showing nylon‐backbone structures to be most effective. The actuator's controllability and speed were demonstrated through static ejection and dynamic interception tasks, including three‐point shot and baseball home run scenarios, while integration with a recognition algorithm enabled rapid perception‐action coupling.

Building on this foundation, we developed the hybrid Jump‐and‐Fly Catbot (JFC), which combines the combustion‐driven soft actuator with electric motors to achieve multimodal terrestrial‐aerial locomotion. JFC demonstrates millisecond‐scale jumping with power density exceeding 3700 W/kg, seamless jump‐to‐flight transitions within 0.1 s, and controlled aerial maneuvers, allowing rapid escape from entrapment and agile navigation in an unstructured environment. In comparison with existing multimodal robotic platforms, JFC uniquely integrates high‐force actuation, precise control, and versatile locomotion for reliable operation in complex environments. Inspired by feline biomechanics, where energy is stored in an arched spine before explosive release, the actuator's asymmetric backbone produces powerful yet controllable bending motions. Together, these results establish a new paradigm for soft robotics, enabling machines that can interact robustly with complex environments by delivering powerful strikes, executing rapid leaps, achieving effective escape, and performing agile maneuvers for reliable operation across terrestrial and aerial domains.

## 4 Methods

### 4.1 Fabrication of the Combustion‐Driven Soft Actuator

The backbone structure, consisting of six semi‐ring‐shaped ribs (diameter: 25 mm; inclination: 80° relative to the central axis), constrains radial balloon expansion. Fabricated via nylon 3D printing (Young's modulus: 1646 MPa), this integrated structure balances toughness and elasticity. The combustion chamber consists of a silicone rubber balloon formed by mixing equal volumes of silicone gels A and B, casting into rectangular molds, and curing at room temperature for 7 h. Final assembly integrates the balloon with the backbone (detailed in Figure S26).

### 4.2 Fabrication of the Gas Supply System

The gas flow control system (premix propane and oxygen) integrates oxygen and propane cylinders housed in a safety cabinet, connected to a flow meter with gas safety alarms. For premixed gas generation, the flow meter regulates valve opening duration to precisely control gas volume. The solenoid valve, igniter, and exhaust check valve are controlled by a microcontroller. Following a single actuation cycle, the solenoid valve opens to allow for a secondary inflation. Upon completion of inflation, the mixture was ignited again to achieve a second actuation. The continuous inflation and ignition system design principle is illustrated in Figure S32.

This study primarily focuses on single actuation to elucidate the coupling mechanisms between combustion actuation and electric motor systems. In specific application scenarios such as rapid extrication, a single actuation was already sufficient to achieve the desired performance. The key to realizing multi‐cycle actuation of the robot lies in the implementation of a portable and untethered energy supply. A portable and untethered energy supply has been achieved in combustion‐driven robots. For instance, Loepfe et al. incorporated nitrous oxide‐propane/butane gas tanks into a jumping soft robot for fully cordless operation [51]; Bartlett et al. integrated butane‐oxygen fuel storage in a 3D‐printed robot to realize untethered jumping [52]; Yang et al. equipped an 88‐mg insect‐scale robot with methanol fuel tanks for autonomous locomotion [53]. These references sufficiently support the technical feasibility of portable power sources. In this work, we focus on the investigation of the hybrid actuation mechanism between combustion and electric drives. In future studies, we plan to integrate such portable energy supply technologies to enable sustained multi‐cycles actuation.

### 4.3 Actuating Experiment

To investigate the influence of premixed oxygen‐propane gas ratios and volumes on combustion actuation, we conducted actuating experiments. The setup (Figure S26) comprises: a soft combustion actuator mounted on a base, an ignition system with battery‐powered booster and micro‐wires, and a gridded calibration board (85 × 150 cm) enabling projectile trajectory tracing. Actuation performance was evaluated through thrown displacement, transient velocity, and acceleration measurements. A high‐speed camera (IDT Motion Pro Y7, 10000 fps) positioned orthogonally to the board captured motion kinematics for quantitative analysis.

### 4.4 Numerical Setup

To further investigate the effect of combustion pressure and backbone materials on the driving performances, the actuation was simulated in ABAQUS. We selected different elastic materials for the backbone (e.g., resin, nylon, and PLA) to comprehensively study the influence of the material on the actuation. The entire body of the actuator was subjected to gravity, and uniformly distributed combustion stress was applied to the balloon's inner surface. The full contact method was employed to describe the interaction characteristics. The physical properties of the actuator and ball in the numerical model are consistent with those observed in the experiment. The detailed parameters of the numerical model are listed in Table 2.

**TABLE 2:** Detail Setups of Numerical Model.

Objects	Setup items	Values
Nylon	Density (kg/m^3^)	1100
	Young's modulus (MPa)	1646
	Poisson's ratio	0.4
Resin	Density (kg/m^3^)	1200
	Young's modulus (MPa)	2400
	Poisson's ratio	0.3
Polylactic acid (PLA)	Density (kg/m^3^)	1250
	Youngs's modulus (MPa)	3500
	Poisson's ratio	0.425
Ball	Density (kg/m^3^)	707
	Youngs's modulus (MPa)	3
	Poisson's ratio	0.3
Mesh	Mesh size (m)	4.167 × 10^−3^
	Number of elements	20689
Assembly	Interaction type	Full contact
	Step time (s)	1

### 4.5 Pressure Test

To investigate the actual pressure of the silicone tube during rapid actuation of the combustion‐driven soft actuator, we conducted two pressure experiments, including an internal air pressure test and an outer wall pressure test. To measure the internal air pressure of the silicone tube during actuation of the actuator, we inserted the barometer sensor into the silicone tube, as shown in Figure S25. The internal air pressure was 41 kPa when *R* = 4, and *A* = 20 mL. As for the combustion‐driven soft actuator outer wall pressure test, the experimental configuration involves positioning the silicone tube on an experimental platform, with pressure sensors precisely attached to the mid‐section of its outer wall for localized pressure monitoring, as illustrated in Figure S21. When the *R* is 4, the pressure is approximately 38 kPa.

### 4.6 The Analysis Method of Actuator Actuation Simulation

The Explicit dynamics analysis method was mainly used to simulate fast dynamic times, such as collisions and combustion. Compared with the implicit dynamics analysis method, the explicit dynamics method was more effective in handling highly nonlinear problems, especially in events that occur within a short period. The equations of motion for the body are integrated using the explicit central difference integration rule,

(1) $$\dot{u}\left(i+\frac{1}{2}\right)=\dot{u}\left(i+\frac{1}{2}\right)+\frac{\Delta t^{\left(i+1\right)}+\Delta t^{\left(i\right)}}{2}{\ddot{u}}^{\left(i\right)}$$

(2) $$u^{\left(i+1\right)}=u^{\left(i\right)}+\Delta t^{\left(i+1\right)}\dot{u}\left(i+\frac{1}{2}\right)$$

where $\dot{u}$ is velocity, and $\ddot{u}$ is acceleration. The superscript (*i*) refers to the increment number. The central difference integration operator is explicit in that the kinematic state can be advanced using known values of ${\dot{u}}^{\left(i-1/2\right)}$ and ${\ddot{u}}^{\left(i\right)}$ from the previous increment.

### 4.7 Fabrication of the Jump‐and‐Fly Catbot

To enable soft robots to traverse discontinuous terrains and execute rapid escapes, we present a combustion‐propulsion hybrid robot (JFC). Its core integrates a combustion‐driven soft actuator with four brushless motors (7.5 cm blades) for thrust generation. An upper‐mounted lithium battery and flight controller support hovering and aerial maneuvers, while a head‐mounted miniature camera facilitates autonomous terrain assessment for identifying landing sites.

### 4.8 Fabrication of the Untethered Combustion‐Driven System

To eliminate entanglement risks from cabled actuation in tree‐traversing tasks, we developed an untethered combustion system for JFC. Figure S19 details this system's architecture: a miniature wireless receiver, Li‐ion battery, voltage booster, electrodes, and remote controller. Remote ignition triggers instantaneous combustion, followed by synchronized rotor spin‐up and immediate flight initiation.

## Author Contributions

All authors discussed the results and commented on the manuscript. Zhiguo He initialized the concept, designed the research, and finalized the manuscript. Hongkuan Ma built the prototypes, conducted quantitative experiments, designed a bio‐inspired robot, designed the Jump‐and‐Fly Catbot, processed the corresponding data, developed the FE model, drew the figures and drafted the manuscript. Yang Yang initiated the concept, developed the prototypes, created the FE model, designed the experiments and contributed to finalizing the draft. Pengcheng Jiao designed the research and edited the final version. Hongliang Ren revised and edited the manuscript.

## Conflicts of Interest

The authors declare no conflicts of interest.

## Supporting information




**Supporting File 1**: advs73427‐sup‐0001‐SuppMat.docx.

**Supporting File 2**: advs73427‐sup‐0002‐SI Movies.zip.

**Supporting File 3**: advs73427‐sup‐0003‐Data.xlsx.

## Data Availability

All data needed to evaluate the conclusions in the article are present in the article and the supplementary materials. Additional data related to this article may be requested from the authors.
