# Supplementary material for: Feline‐Inspired Robot Enabled by Combustion‐Driven Actuators for Agile Motion and High‐Payload Obstacle Traversal
Source: Adv Sci (Weinh). 2025 Dec 26;13(18):e19885. doi: 10.1002/advs.202519885 (PMC13042569; doi:10.1002/advs.202519885)
Supplement: Supplementary file 1 — Supporting File 1: advs73427‐sup‐0001‐SuppMat.docx. [file ADVS-13-e19885-s002.docx]

Supplementary Information for

**Feline-Inspired Robot enabled by Combustion-Driven Actuators for Agile Motion and High-Payload Obstacle Traversal**

Hongkuan Ma *et al*.

*Corresponding author. Email: hezhiguo@zju.edu.cn and pjiao@zju.edu.cn

**This PDF file includes:**

Supplementary Text 1 to 11

Figures S1 to S33

Tables S1 to S2

Movies S1 to S10

**Other Supplementary Materials for this manuscript include the following:**

Movies S1 to S10

Supplementary Text

**Supplementary Text 1: The Reaction Mechanism of Combustion**

The reaction mechanism of combustion is:

$$\begin{aligned} C_{3}H_{8}+5O_{2}\underset{\to}{\mathrm{Ignition}}3\mathrm{CO}_{2}+4H_{2}O+heat\#\left( \text{1} \right) \end{aligned}$$

The exergonic oxidation of oxygen and propane ($R$ = 5) generates carbon dioxide, water vapor, and substantial thermal energy. It should be noted that some unwanted byproducts (e.g., soot, $\mathrm{CO}_{X}$ compounds, $\mathrm{NO}_{X}$ compounds) which lead to insufficient reaction are produced during the reaction process, due to the propane and oxygen involved in the reaction are not pure. Therefore, it is necessary to study how the ratio of propane to oxygen influences the actuating performances of combustion-enabled powerful soft actuator.

**Supplementary Text 2: The Relationship Between the Detonation Velocity and Pressure of the Gas Mixture**

Within the initial pressure range of 0.01–10 MPa, the relationship between detonation velocity and pressure for certain gaseous mixtures exhibits the following functional form:

$$\begin{aligned} D_{p}=D_{p_{0}}+\beta\lg\left( \frac{P}{P_{0}} \right)\#\left( \text{2} \right) \end{aligned}$$

Here, $D_{p}$ and $D_{p_{0}}$ represent the detonation velocities under pressures $P$ and $P_{0}$, respectively. $\beta$ is a dimensionless coefficient whose value equals the increment in detonation velocity corresponding to a tenfold increase in pressure.

**Supplementary Text 3: The Motion Performance of the Actuator in Baseball Home Run**

From 0 s to 0.26 s, baseball is in free-fall motion, with a velocity of 0 m/s in the horizontal direction, and the maximum downward velocity is 3.13 m/s. At 0.26 s, as the baseball approaches actuator and the combustion actuate, and then actuator accurately hits the baseball. The combustion-driven soft actuator has provided baseball with powerful contact and upward slanting thrust that induces a huge and rapid change in the locomotion characteristics of the baseball.

The velocity in the horizontal direction reaches 2 m/s at 0.4 s (Figure. S6), and the instantaneous horizontal acceleration reaches 20 ${m/s}^{2}$. The upward velocity reaches 2.9 m/s at 0.32 s (Figure. S6), and the instantaneous upward acceleration reaches 105.5 ${m/s}^{2}$. It should be noted that the instantaneous synthetic acceleration of a baseball that is hit by combustion-driven soft actuator reaches 107.38 ${m/s}^{2}$.

**Supplementary Text 4: The Vertical Sensing-actuating Demonstration of the Actuator**

To validate that the combustion-driven soft actuator has the abilities of precise controllability and optical sensing-actuating, we have conducted the sensing-actuating experiment, including precision recognition and actuating demonstrations in the vertical direction and horizontal direction. In the vertical sensing-actuating demonstration, the micro-camera is mounted on the bottom of the actuator and shoots upwards, as shown in Figure. S22. As the ball falls, the micro-camera can record the motion of the ball and analyze the changing of ball’s diameter in the image. When the diameter of the ball, captured by the micro-camera, reaches a threshold, the computer transmits a signal to the microcontroller, which controls the actuator’s automatic actuating to hit the ball accurately. The results of the demonstration experiment are shown in Figure. S9. The ball falls freely at 0 s, and at 0.3 s, the micro-camera recognizes that the diameter of the ball has reached the threshold and transmits signals to the microcontroller. At 0.36 s, the actuator receives the signal and hits the ball precisely. The demonstration has been featured in Movie S4.

**Supplementary Text 5: The Jumping Motion of the Jump-and-Fly Catbot**

The experimental configuration for the Jump-and-Fly Catbot’s jumping motion is illustrated in Figure. S10. A single-chip microcontroller functions as the control system and interfaces with relays. These relays connect to the remote controller and spark igniter, respectively. The microcontroller quantitatively regulates the temporal interval between combustion-driven actuation and rotor-driven actuation. Motion performance characteristics of the Jump-and-Fly Catbot are presented in Figures. S11 and S12. Figure. S12B depicts the robot’s motion trajectories between 0 s and 0.2 s under varying power densities. To examine the influence of takeoff angle on motion characteristics, launch angles of 5°, 10°, 20°, and 30° were investigated. The maximum jumping distance was achieved at a 10° takeoff angle, as demonstrated in Figure. S12C. Power density was evaluated across different premixed gas ratios and gas volumes. A peak power density of 3730 W/kg was attained at a propane-to-oxygen ratio of 1:5 with 25 ml gas volume, as shown in Figure. S12D. The maximum acceleration during jumping reached 800 m/s². Figure. S12F illustrates the time evolution of the robot’s pitch angle during jumping under different power densities.

**Supplementary Text 6. The Flight Motion of the Jump-and-Fly Catbot**

The Jump-and-Fly Catbot is capable of multiple flight modalities, including upward motion, forward motion, downward motion, straight motion, steering motion, and zigzag motion, as demonstrated in Figure. S14A, B, C, and D. Corresponding motion trajectories and velocity are presented in Figure. S14E, F, and G. We further conducted a quantitative comparison between experimentally measured flight parameters (thrust, pitch angle, acceleration, and power) and calibrated reference values, revealing deviations within a 20% error margin.

**Supplementary Text 7: The Components of the Gas Flow Control System**

The gas flow control system consists of oxygen cylinders, propane cylinders, a flow meter, and gas safety alarms, as shown in Figure. S20. The oxygen and propane cylinders are placed in a safety cabinet and connected to the flow meter. When premixed gas is required, the flow meter is opened to control the opening time of the gas valve to control the gas volume accurately.

**Supplementary Test 8: The Jumping Motion of the Combustion-driven Catbot**

Figure. S23C shows the experimental setups of the combustion-driven Catbot jumping upwards over long distances. The length of the combustion-driven Catbot in a curled-up state is 17.5 cm, the height of the initial launching platform is 30 cm, and the height of the landing platform is 40 cm. The distance between the initial platform and the landing platform is 20 cm, and the jumping displacement is about 38.81 cm (approximately 2.22 times the body length, as shown in movie S5). When the combustion is actuated, the balloon expands rapidly, and the interaction between the balloon and the backbone is full contact. The head of the combustion-driven Catbot performs a fast bending motion due to asymmetric bending stress, and the rear feet of Catbot also tend to perform bending motion in the absence of the bottom fixation restriction. The launch platform provides a reaction force that enables combustion-driven robots to jump rapidly. Figure. S23D, E, and F present the trajectory of combustion-driven Catbot jumping locomotion, the relationship between horizontal velocity and time, and the relationship between vertical velocity and time, respectively. The horizontal velocity of the combustion-driven Catbot is up to 1.1 m/s (6.1 BLPS) at 0.05 s, and the horizontal instantaneous acceleration is up to 22 m/s^2^. Regarding upward motion, the velocity is up to 0.83 m/s (about 4.74 BLPS), and the instantaneous acceleration is up to 11.1 m/s^2^. The weight of the combustion-driven Catbot is 126 g, and this Catbot has demonstrated a considerable payload capacity of 40 times its body weight. The photographs of the long-distance upward jumping experiment are shown in Figure. S23G. Furthermore, the Catbot’s motion performances on a flat surface have been shown in Figure. S24.

The Catbot’s maximum velocity and acceleration, reaching 7.7 BLPS and 30 m/s^2^, respectively, are similar to the locomotion of a natural cat (The instantaneous speed is about 8 BLPS, and the instantaneous acceleration is up to 20 m/s^2^). Furthermore, in contrast to other cat-inspired robots, this combustion-driven Catbot accelerates more rapidly and can jump upward for long distances (more than 2 times its body length) with multiple complex motions, offering a promising avenue for bionic soft robots to overcome obstacles with agility and realize multiple response motions under a single driving mechanism.

**Supplementary Text 9: Pressure Governing Equations and Empirical Equations During Combustion**

The equation of state for an ideal gal is given.

$$\begin{aligned} PV=nRT\#\left( \text{3} \right) \end{aligned}$$

where $P$ is pressure, $T$ is temperature, $V$ is the volume of silicone tube, $n$ is the number of moles of gas species, and $R$ is the universal gas constant. The number of moles of gas species has been shown.

$$\begin{aligned} n=\frac{m}{\sum y_{i}M_{i}}\#\left( \text{4} \right) \end{aligned}$$

where $m$ is total mole number, $y_{i}$ is mole fraction of components, $M_{i}$ is mole mass. The ratio and amount of premixed gas affect the $m$ and $y_{i}$, respectively.

When combustion is initiated instantaneously, the volume of the silicone tube can be approximated as constant. We can express the equation of state in different form with time *t*.

$$\begin{aligned} \frac{1}{P}\frac{dP}{dt}=\frac{1}{n}\frac{dn}{dt}+\frac{1}{T}\frac{dT}{dt}\#\left( \text{5} \right) \end{aligned}$$

For the latent variables of $n$ and $T$ in combustion, we apply equations respectively.

$$\begin{aligned} \frac{dn}{dt}=\sum_{k\in R_{n}} I_{k}N_{k}\dot{\omega}_{k}\#\left( \text{6} \right) \end{aligned}$$

$$\begin{aligned} \frac{d}{dt}\left( \sum_{i\in S_{p}} \left\{ mC_{v} \right\}_{i}T_{i} \right)=\sum_{k\in R_{n}} \tilde{m}_{k}\varepsilon_{k}H_{k}\dot{\omega}_{k}-\dot{Q}_{loss}\#\left( \text{7} \right) \end{aligned}$$

where $N_{k}$ is the amount of gas release from reaction, $R_{n}$ is a set of reactions, $I_{k}$ is the sign of gas generation, $\dot{\omega}_{k}$ is the reaction rate, $S_{p}$ is a set of species, $m$ is the mass, $C_{v}$ is the specific heat capacity, $\tilde{m}_{k}$ is the mass of reactants participating in the reaction, $\varepsilon_{k}$ is the combustion efficiency, $H_{k}$ is the heat of reaction, and $\dot{Q}_{loss}$ is the heat loss.

Based on internal pressure experiments, we derive the following empirical formula for calculating pressure within the silicone tube.

$$\begin{aligned} P=\alpha A+\beta R-\gamma R^{2}+\delta AR-\mu\#\left( \text{8} \right) \end{aligned}$$

where $\alpha$, $\beta$, $\gamma$, $\delta$, $\mu$ are gas volume linear coefficient which is 1.16 kPa/ml, ratio linear coefficient which is 26.43 kPa, ratio quadratic term coefficient which is 3.1 kPa, ratio and gas volume coupling coefficient which is 0.08 kPa/ml, constant which is 46.4 kPa, respectively. The fitted surface model is presented in Figure. S27, exhibiting a goodness-of-fit ($R^{2}$) of 0.988, a root means square error (RMSE) of 1.52, and a mean absolute percentage error (MAPE) of 3.63%.

**Supplementary Text 10: Multiple Actuation Experiment**

We conducted 30 actuations under the conditions of $A$ = 20 ml and $R$ = 4. By calculating the peak acceleration of the ball within 10 ms and the peak output force of the actuator, we determined that the object achieved an average acceleration of 3260 ${m/s}^{2}$, while the actuator delivered an average output force of approximately 65.04 N (about 72 times its self-weight), with a relative standard deviation consistently below 5%, shown in Figure S33.

Additionally, the actuator has been equipped with automatic inflation and actuation capabilities, as described in the Methods section and illustrated in Figure S32, with the relevant content now highlighted. We integrated this combustion-driven actuator with electric motor systems to develop the Jump-and-Fly Catbot, which is capable of navigating unstructured terrains and demonstrating robust escape behaviors, such as rapid extrication and takeoff.

This study primarily focuses on single actuation to elucidate the coupling mechanisms between combustion actuation and electric motor systems. In specific application scenarios such as rapid extrication, a single actuation is already sufficient to achieve the desired performance. Nevertheless, we fully agree with the reviewer’s valuable suggestion regarding the importance of performance under repeated actuation cycles. The key to realizing multi-cycle actuation of the robot lies in the implementation of a portable and untethered energy supply. Portable and untethered energy supply has been achieved in combustion-driven robots. For instance, Loepfe et al. incorporated nitrous oxide-propane/butane gas tanks into a jumping soft robot for fully cordless operation;^[51]^ Bartlett et al. integrated butane-oxygen fuel storage in a 3D-printed robot to realize untethered jumping;^[52]^ Yang et al. equipped an 88-mg insect-scale robot with methanol fuel tanks for autonomous locomotion.^[53]^ These references sufficiently support the technical feasibility of portable power sources. In this work, we focus on the investigation of the hybrid actuation mechanism between combustion and electric drives. In future studies, we plan to integrate such portable energy supply technologies to enable sustained multi-cycles actuation.

**Supplementary Text 11: The structural design of the combustion-driven soft actuator**

Numerical simulations were conducted with 3, 6, and 10 semicircular ribs. Under an internal combustion pressure of 40 kPa, the actuator with only 3 ribs exhibited significant structural damage at 0.005 s, as shown in Figure S29. We analyzed the displacement and stress at the top region of the actuator (Figure S30). The results show that 3 ribs provide insufficient constraint for balloon expansion, whereas 10 ribs overly restrict it. In comparison, the actuator with 6 ribs demonstrates optimal driving performance, achieving a stress value of up to 0.52 MPa.

We further investigated the effect of rib diameter by testing diameters of 20 mm, 25 mm, and 30 mm under the same internal pressure. As shown in Figure S31, smaller rib diameters led to higher output force under identical combustion conditions. In addition, the influence of backbone material was examined (Figure 3). Among the materials tested, the nylon backbone provided the highest driving force under the same combustion pressure.

Figure. S1.


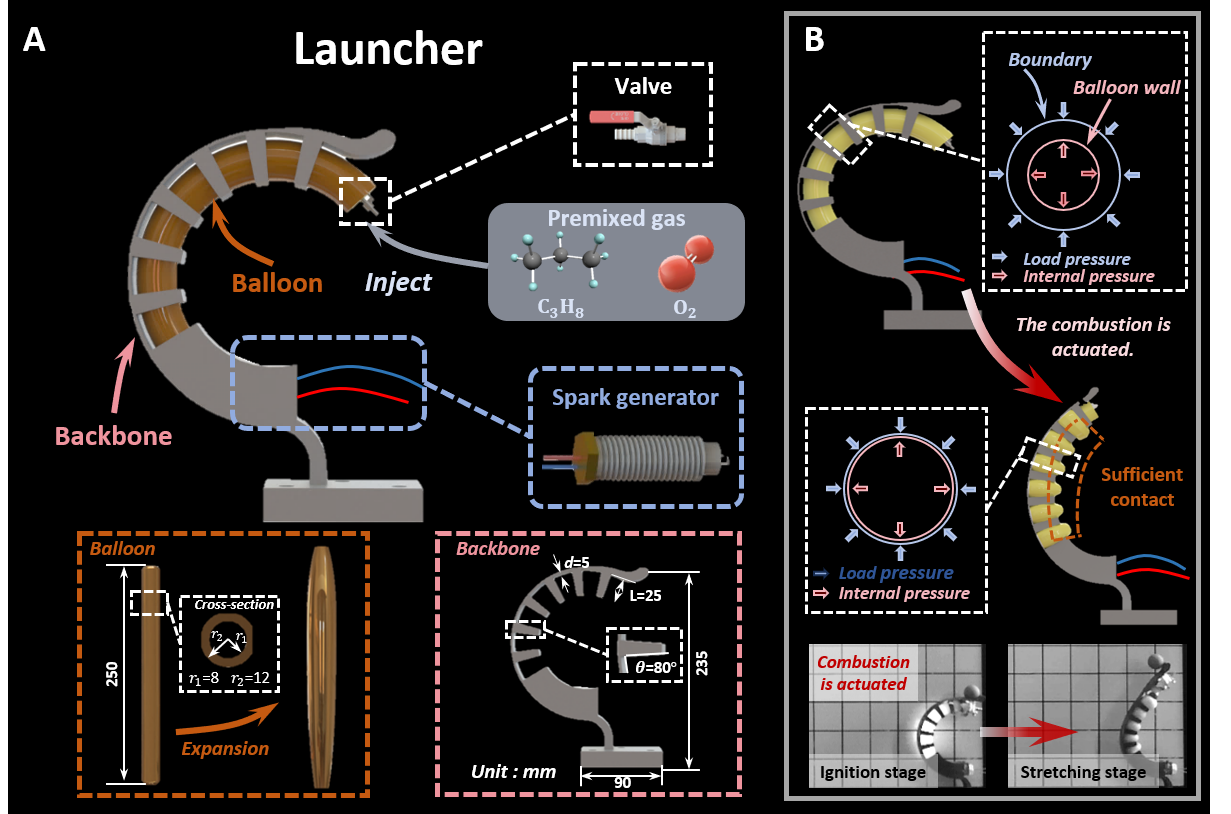


**Figure. S1.** Illustrative demonstration of the combustion-driven powerful soft actuator. (A) The detailed dimensions and structure of the actuator. (B) The driving mechanism of the actuator.

Figure. S2.


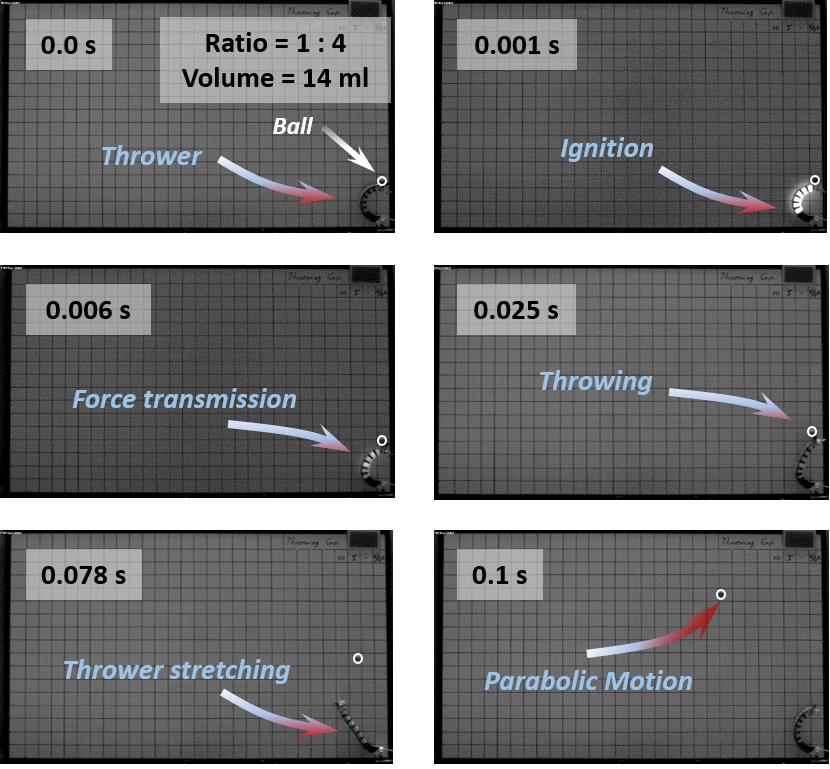


**Figure. S2.** The photograph of actuating test. The combustion is actuated at 0.001 s, and then combustion force is transmitting. At 0.025 s, the object (small ball) has been thrown, and the trajectory of ball’s motion is a parabola.

Figure. S3.


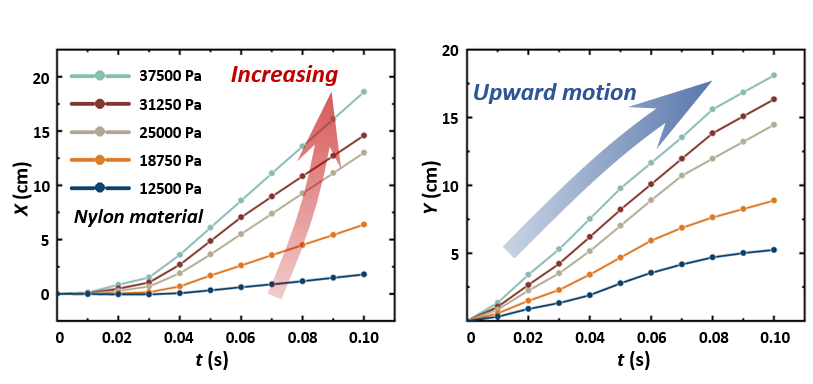


**Figure. S3.** The trajectory of the ball in the horizontal direction and vertical direction versus time. When the material of backbone is nylon, the displacement of the ball’s motion shows a positive correlation with combustion pressure.

Figure. S4.


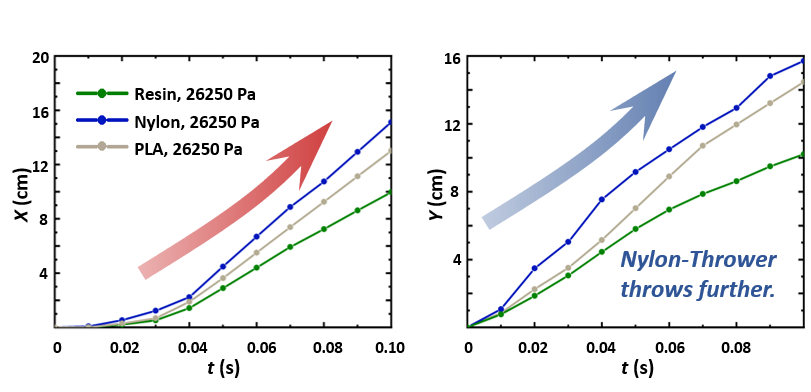


**Figure. S4.** The trajectory of the ball as the material of the backbone is different.

Figure. S5.


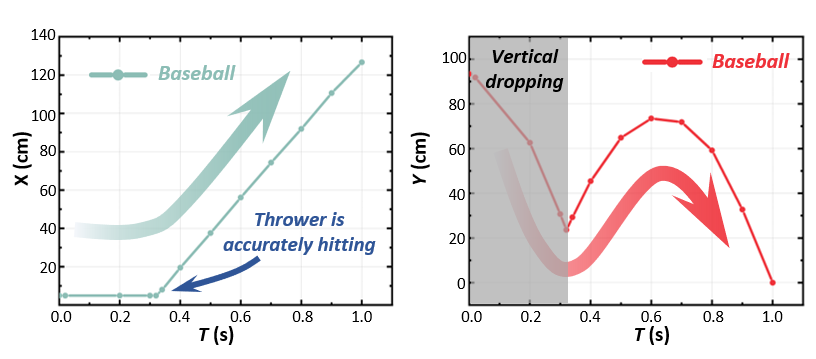


**Figure. S5.** The trajectory of the ball in the horizontal direction and vertical direction versus time, in baseball home run demonstration.

Figure. S6.


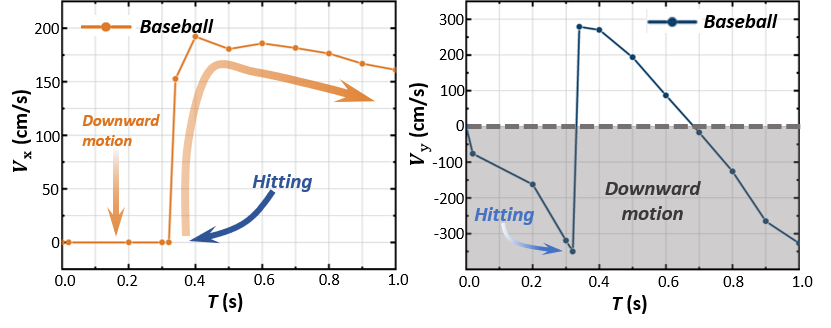


**Figure. S6.** The velocity of the baseball in the horizontal and vertical directions, respectively, in baseball home run demonstration.

Figure. S7.


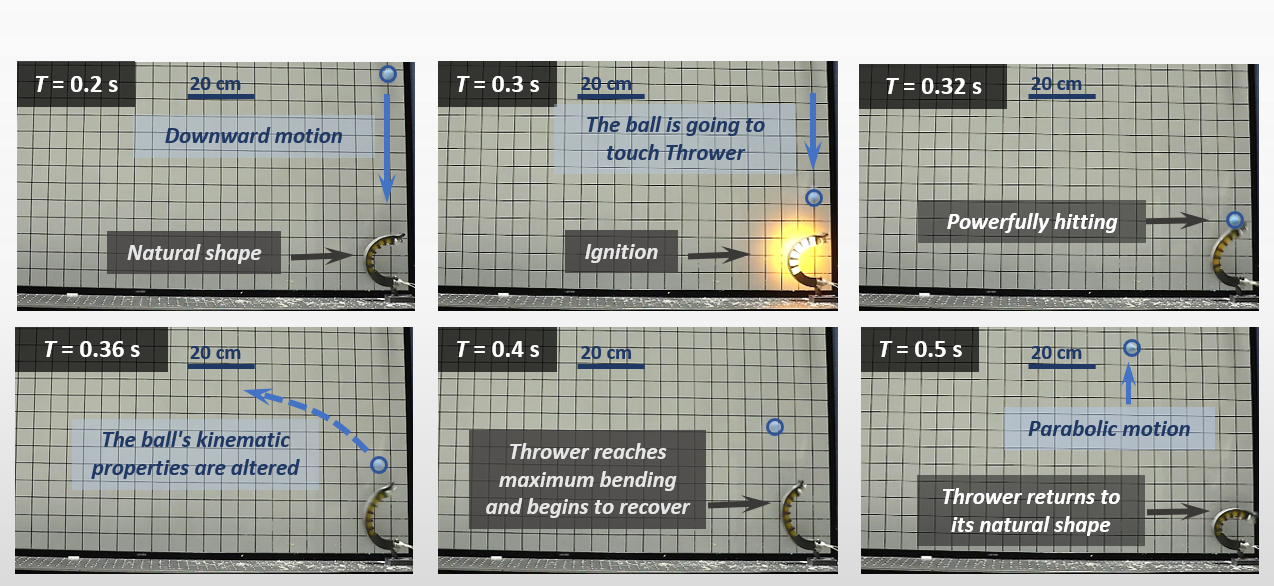


**Figure. S7**. The photographs of the baseball home run demonstration.

Figure. S8.


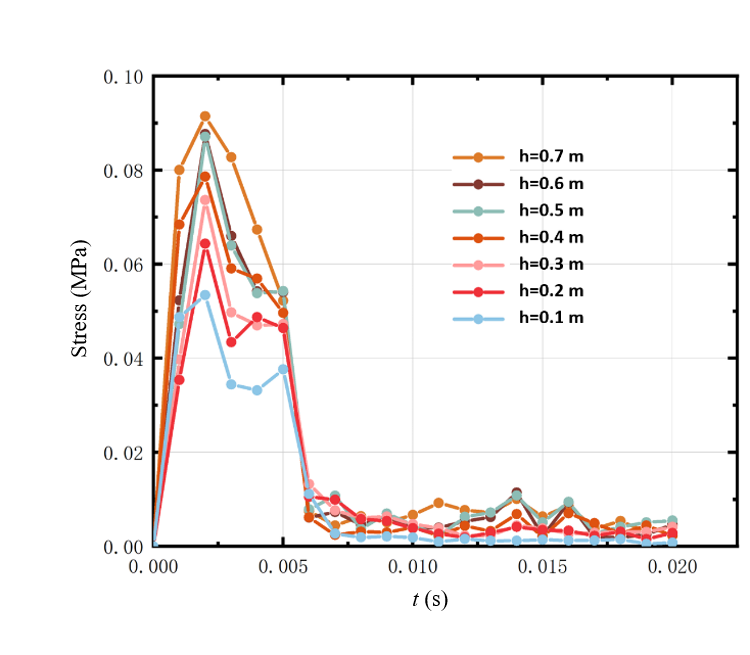


**Figure. S8.** The stress on the contact region of the combustion-driven soft actuator in baseball home run demo, stress is positively correlated with the height of descent of the ball.

Figure. S9.


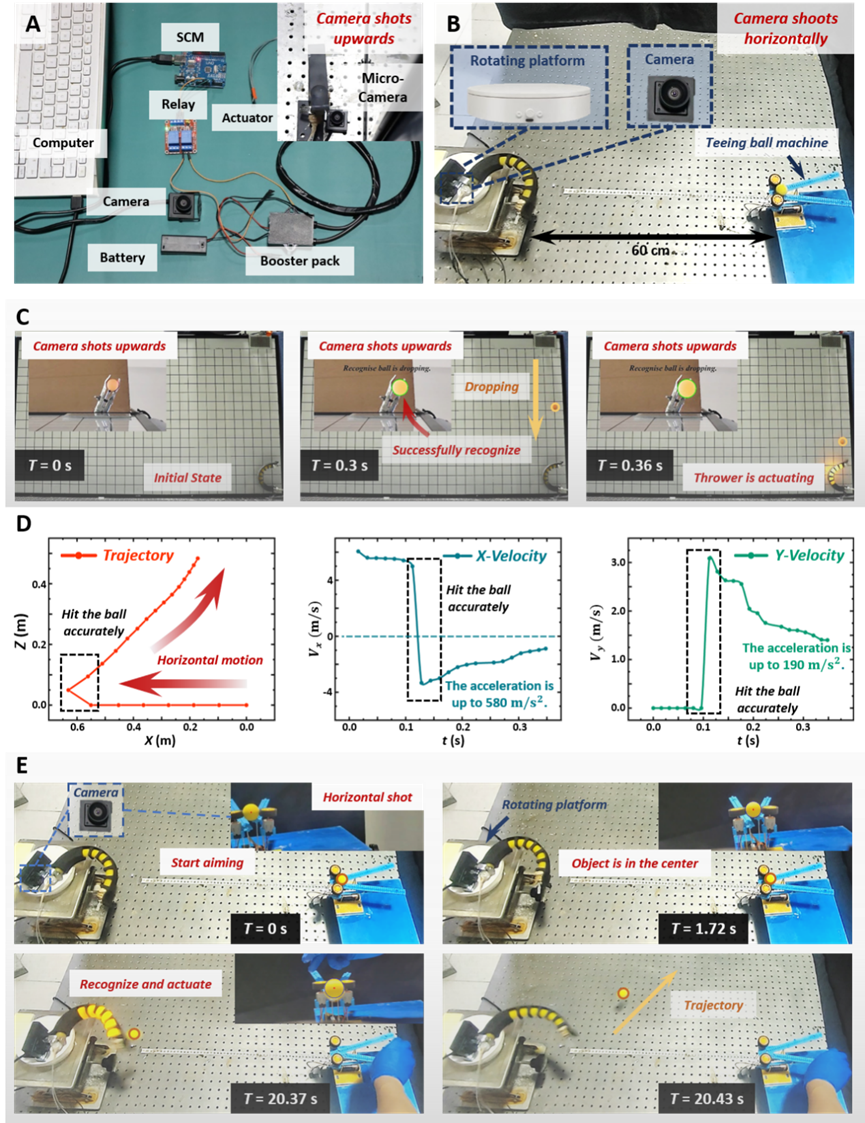


**Figure. S9.** The sensing actuation experiment of the combustion-driven soft actuator. (A) The setup of the vertical actuating experiment. (B) The setup of the horizontal sensing actuation experiment. (C) The photographs of the vertical sensing actuating experiment. (D) The motion trajectory and velocity of the ball in horizontal sensing actuating experiment. (E) The photographs of the horizontal sensing actuating experiment.

Figure. S10.


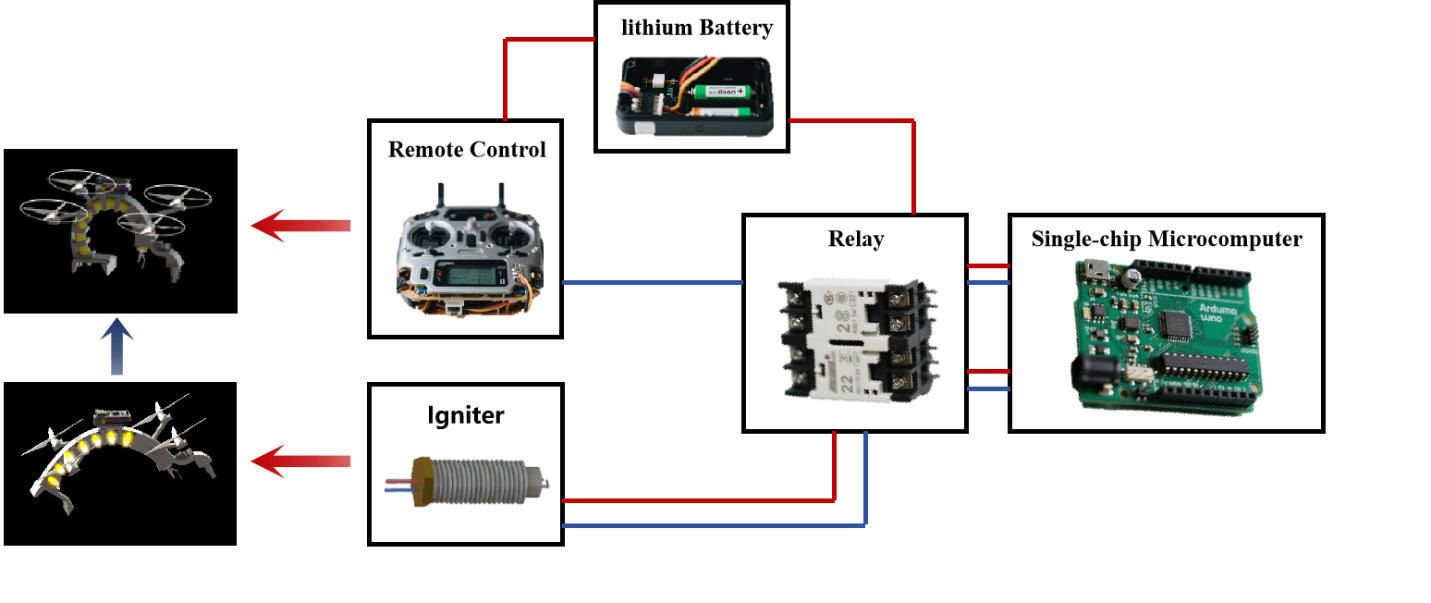


**Figure. S10.** The motion control system of the Jump-and-Fly Catbot.

Figure. S11.


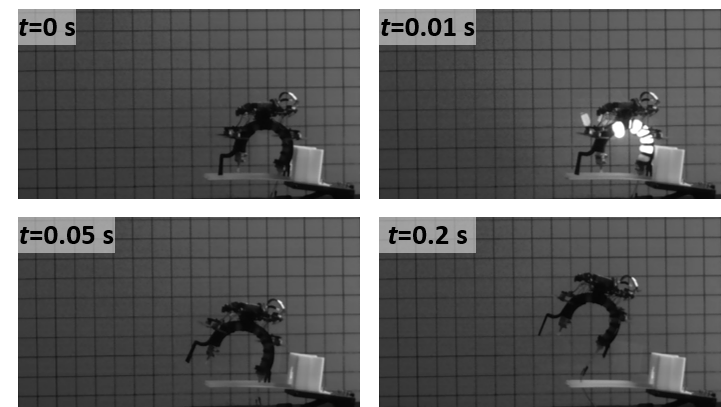


**Figure. S11.** The photographs of the Jump-and-Fly Catbot’s motion.

Figure. S12.


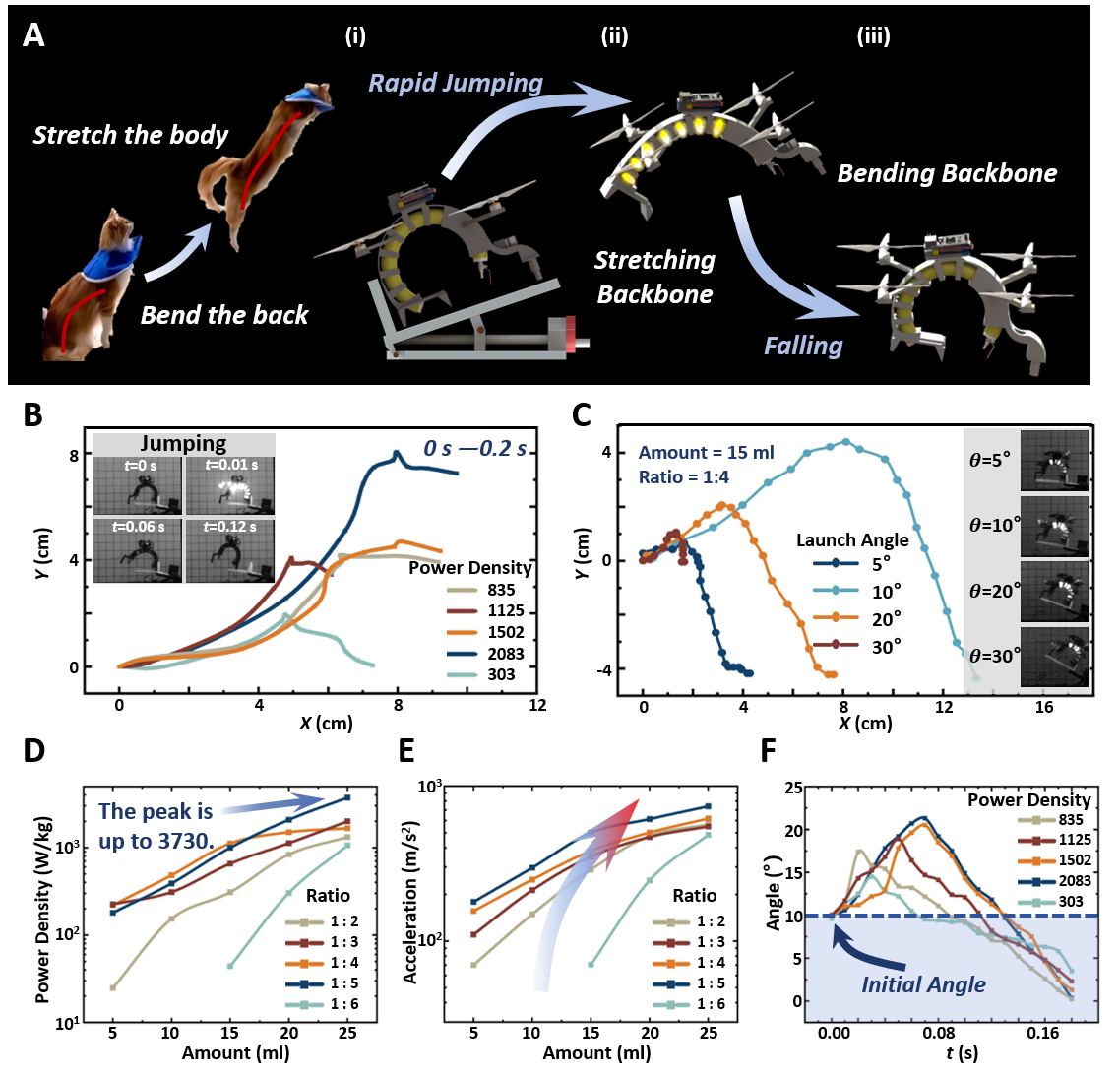


**Figure. S12.** The motion performance of the Jump-and-Fly Catbot. (A) The locomotion mechanism of the Jump-and-Fly Catbot. (B) The trajectory of the Jump-and-Fly Catbot with different power density. (C) The trajectory of the Jump-and-Fly Catbot with different launch angle. When the launch angle is 10°, the Jump-and-Fly Catbot demonstrates excellent motion performance. (D) The power density of the Jump-and-Fly Catbot. When the ratio of propane to oxygen is 1:5 and the gas amount is 25 ml, the power density is up to 3730 W/kg. (E) The instant acceleration of the Jump-and-Fly Catbot is up to 800 ${m/s}^{2}$. (F) The pitch angle of the Jump-and-Fly Catbot with different power density.

Figure. S13.


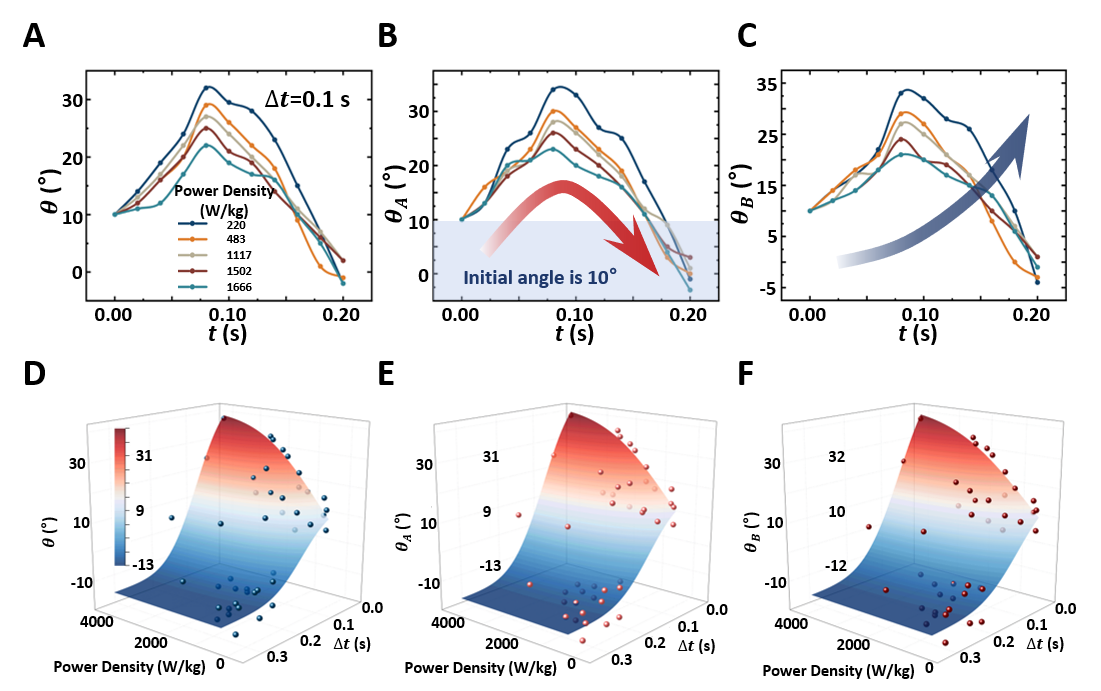


**Figure. S13.** The pitch angle at transition between jumping and flying motions. (A) The Jump-and-Fly Catbot overall pitch angle at the transition between jumping and flying with different power density. (B) The elevation angle of the front side rotor at the transition between jumping and flying with different power density. (C) The elevation angle of the rear side rotor at the transition between jumping and flying with different power density. (D), (E), and (F) show the maximum angles of overall robot elevation, front side rotor elevation, and rear side rotor elevation at different power densities and interval times.

Figure. S14.


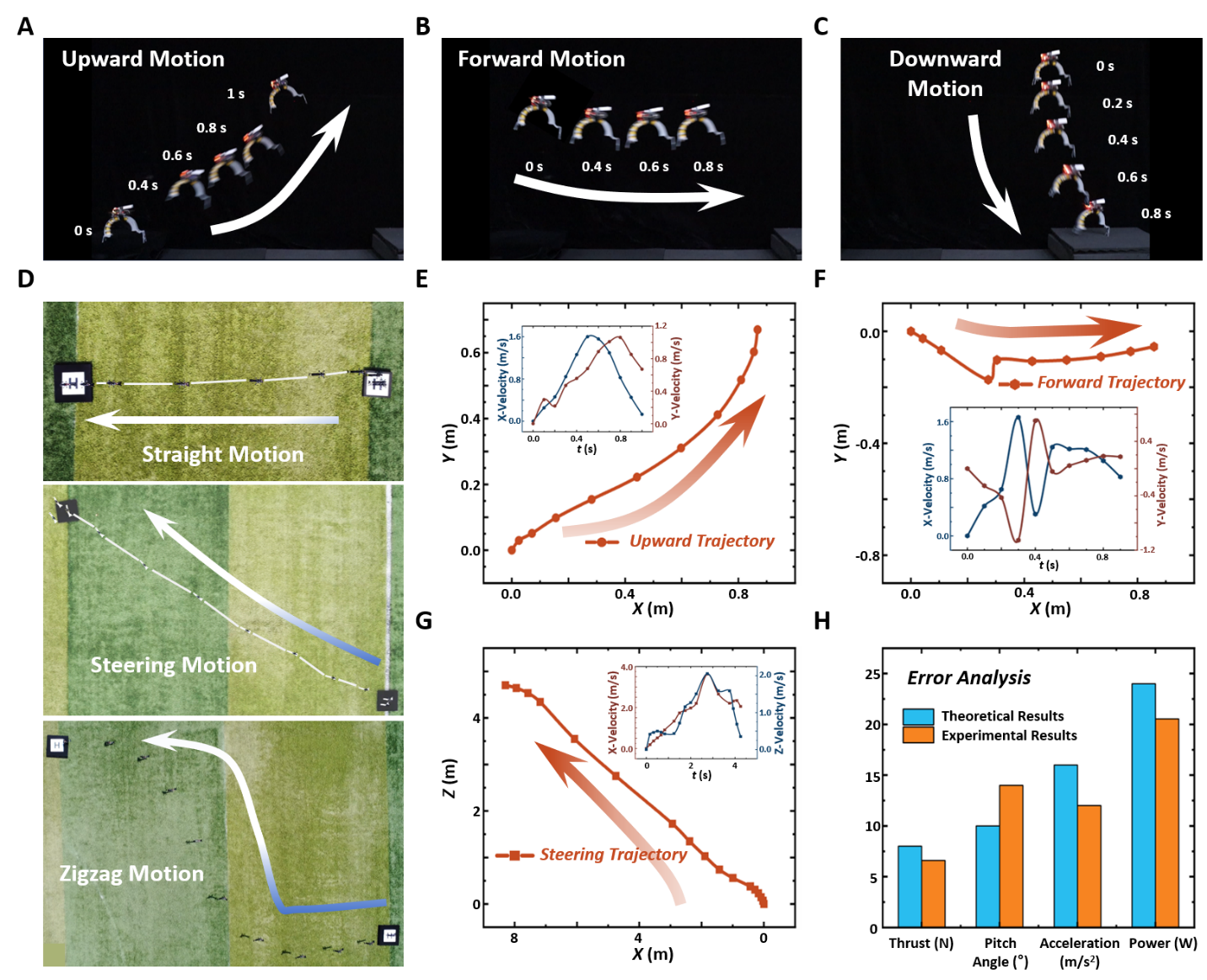


**Figure. S14.** The flight motion of the Jump-and-Fly Catbot. (A) Upward motion. (B) Forward motion. (C) Downward motion. (D) Straight motion, steering motion, and zigzag motion. (E) The trajectory of the Jump-and-Fly Catbot’s upward motion. (F) The trajectory of the Jump-and-Fly Catbot’s forward motion. (G) The trajectory of the Jump-and-Fly Catbot’s steering motion. (H) The error analysis between experimental results and theoretical results, including thrust, pitch angle, acceleration and power.

Figure. S15.


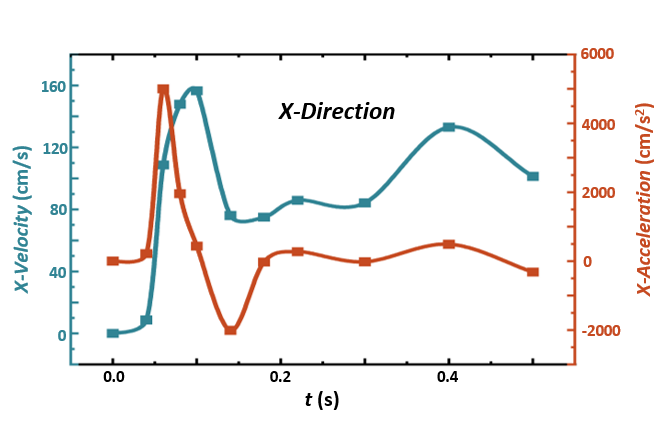


**Figure. S15.** The velocity and acceleration of the Jump-and-Fly Catbot in the X-direction within 0.5 s.

Figure. S16.


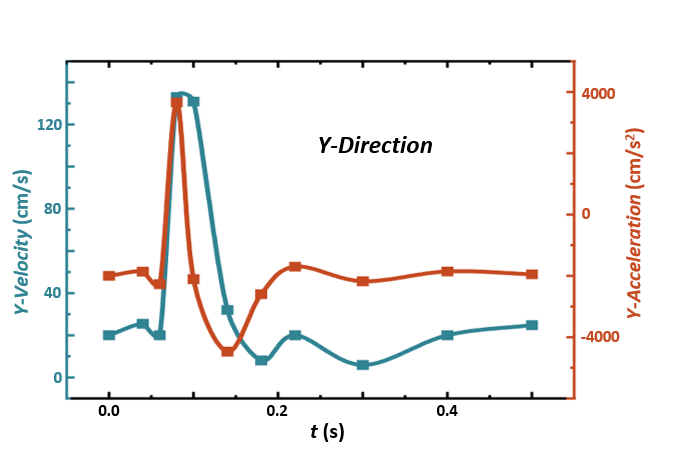


**Figure. S16.** The velocity and acceleration of the Jump-and-Fly Catbot in the Y-direction within 0.5 s.

Figure. S17.


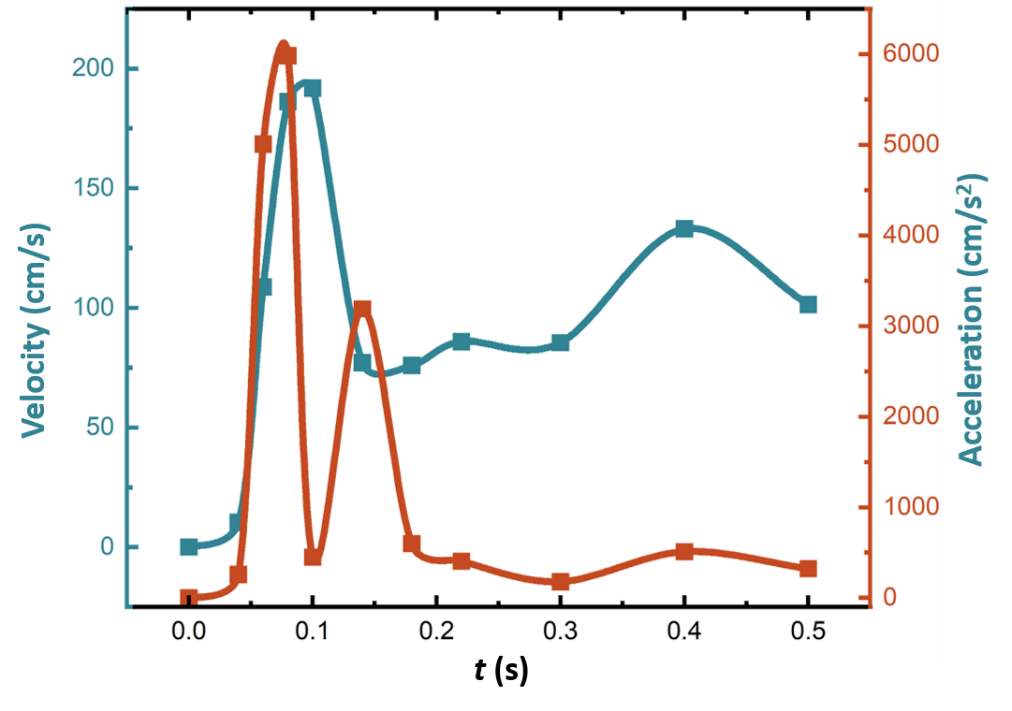


**Figure. S17.** The resultant velocity and acceleration of the Jump-and-Fly Catbot.

Figure. S18.


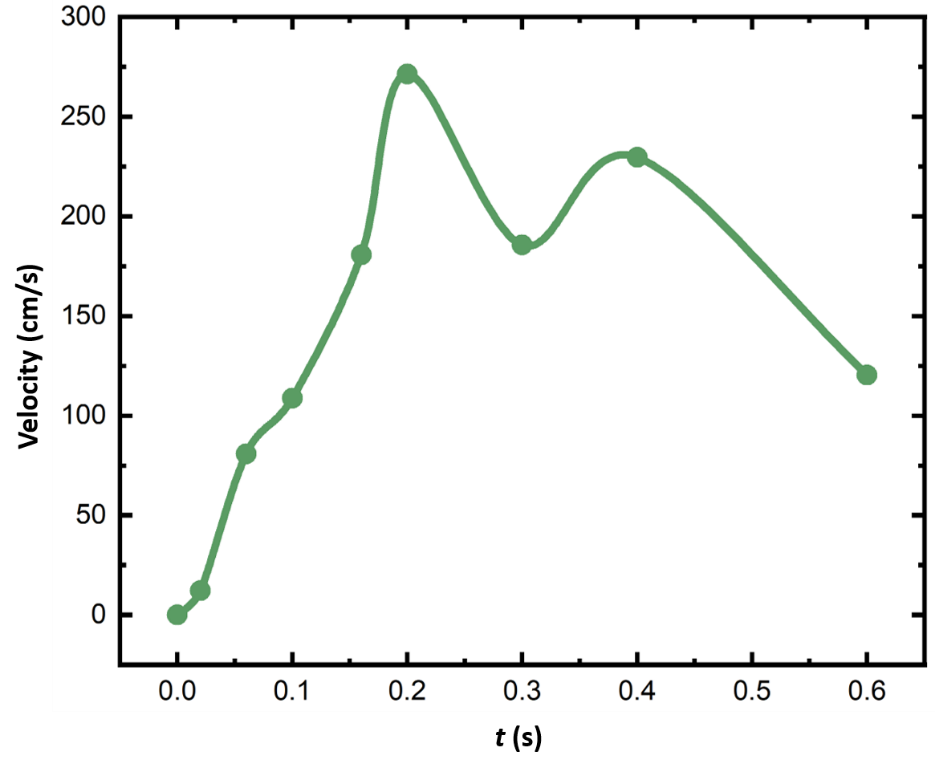


**Figure. S18.** The resultant velocity Jump-and-Fly Catbot within 0.6 s.

Figure. S19.


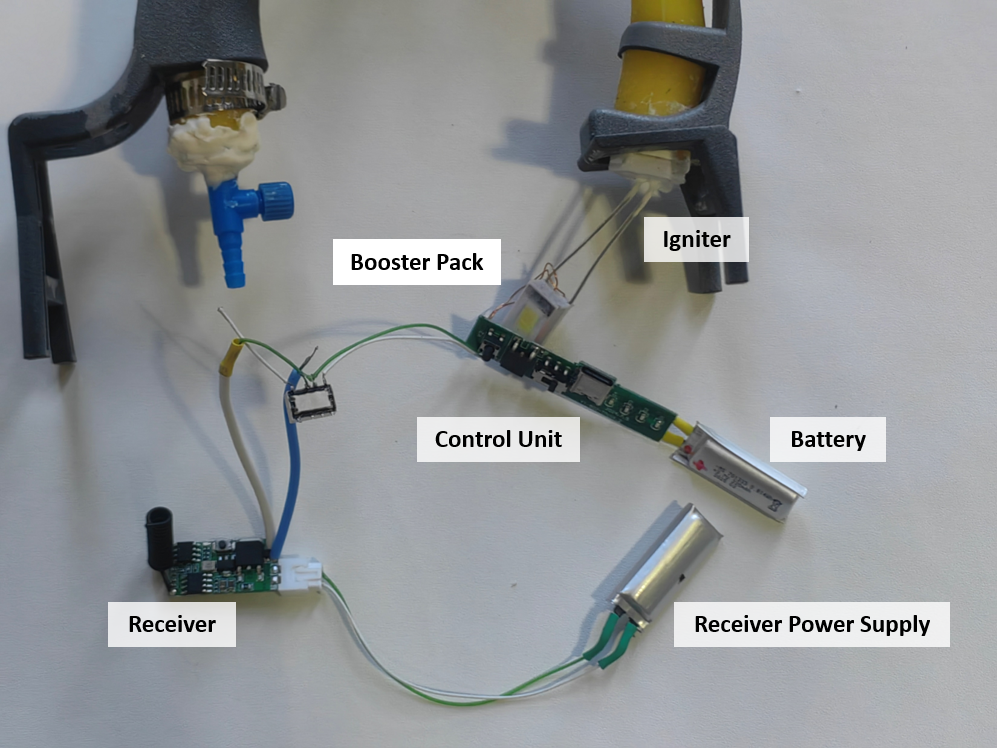


**Figure. S19.** The fabrication of the untethered combustion-driven system, including receiver, control unit, booster pack, battery, receiver power supply, and igniter.

Figure. S20.


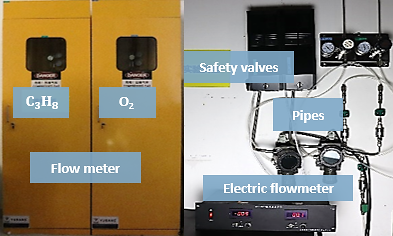


**Figure. S20.** The details of the gas flow control system, including oxygen cylinder, propane cylinder, safety valve, electric flowmeter.

Figure. S21.


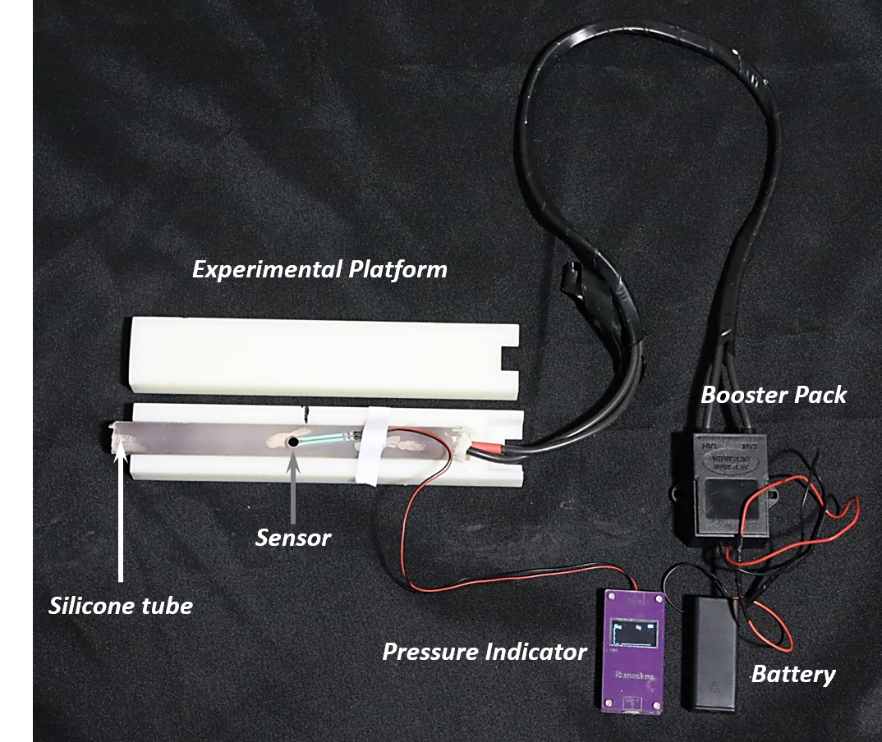


**Figure. S21.** The experimental setup of the combustion-driven soft actuator external surface pressure test.

Figure. S22.


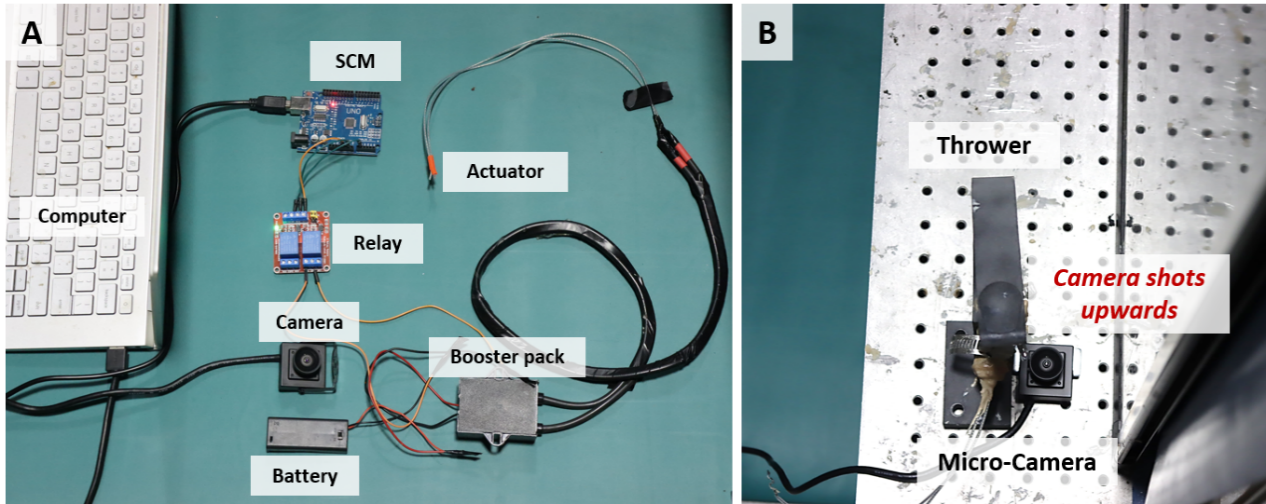


**Figure. S22.** The experimental setup of the robot’s sensing and driving demo. (A) The system includes a computer, a single-chip microcomputer, a relay, a micro-camera, a battery, a booster pack, and an actuator. (B) The micro-camera is mounted on the bottom of the actuator, and the camera shots upwards.

Figure. S23.


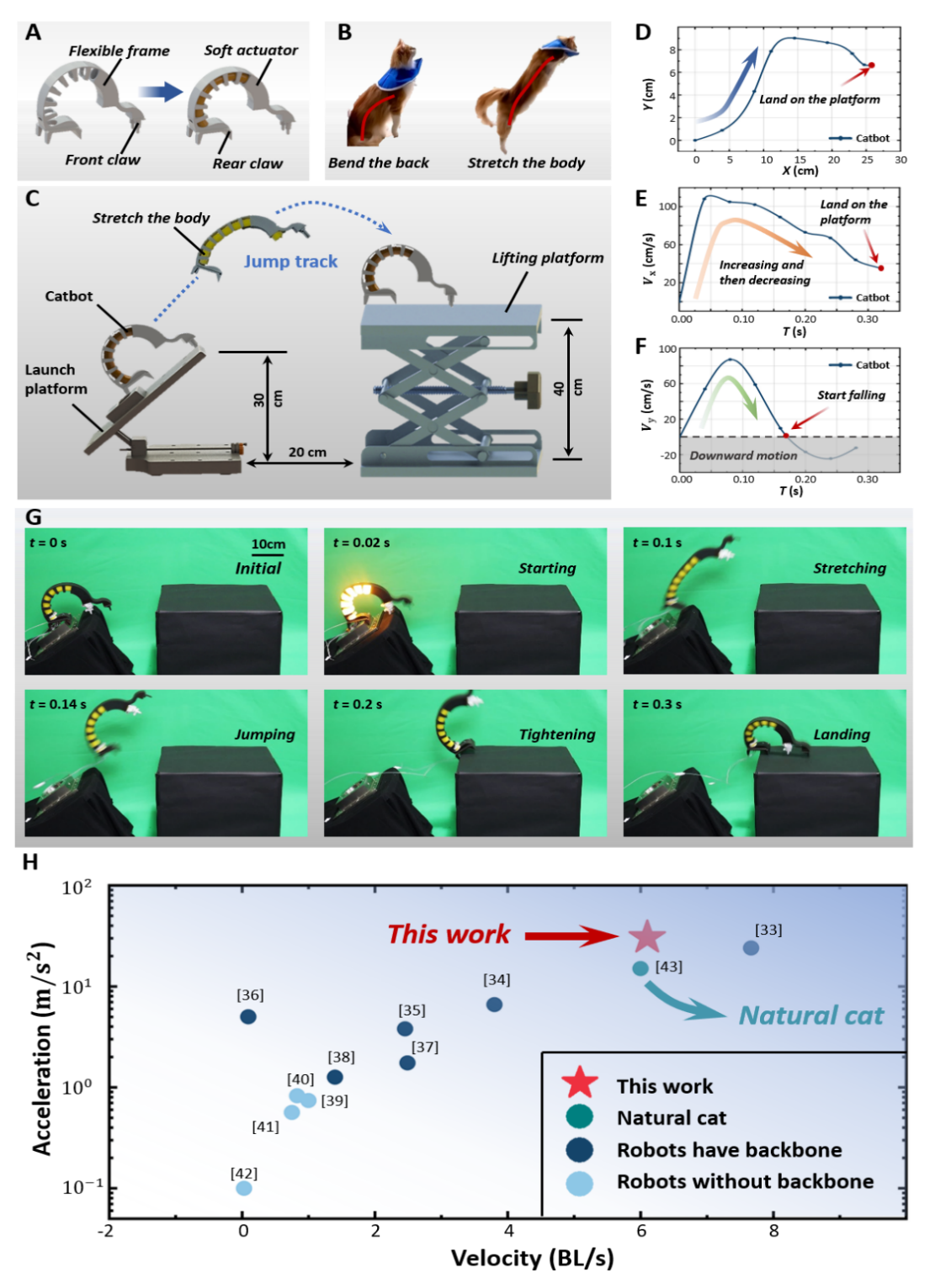


**Figure. S23.** The design principle of the Catbot and the jumping test. (A) The structure of the Catbot. (B) The bionic mechanisms of Catbot. (C) The experimental setup of the jumping test. (D) The jumping trajectory of the Catbot. (E) and (F) show the velocity of the Catbot in the horizontal and vertical directions. (G) The photographs of the jumping experiment.

Figure. S24.


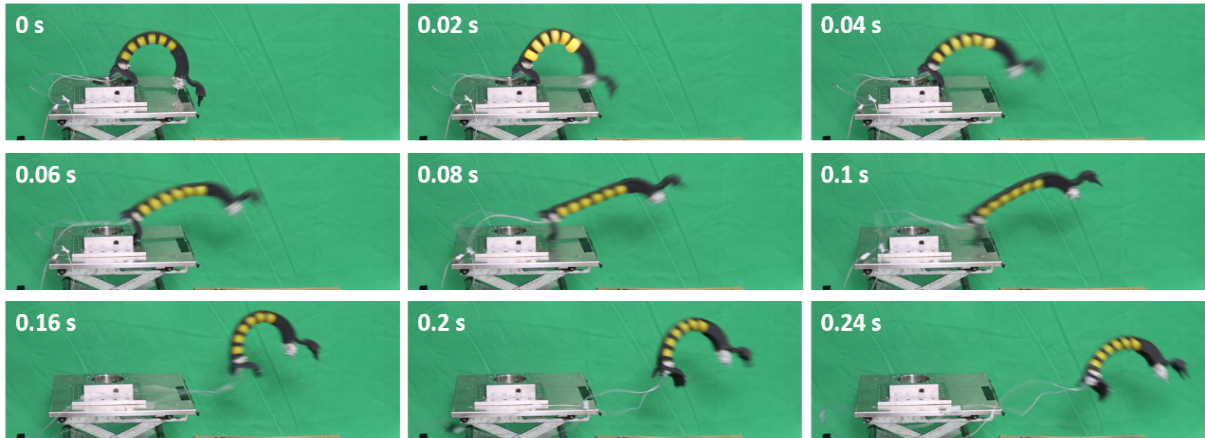


**Figure. S24.** The motion performance of the catbot on a flat surface.

Figure. S25.

**
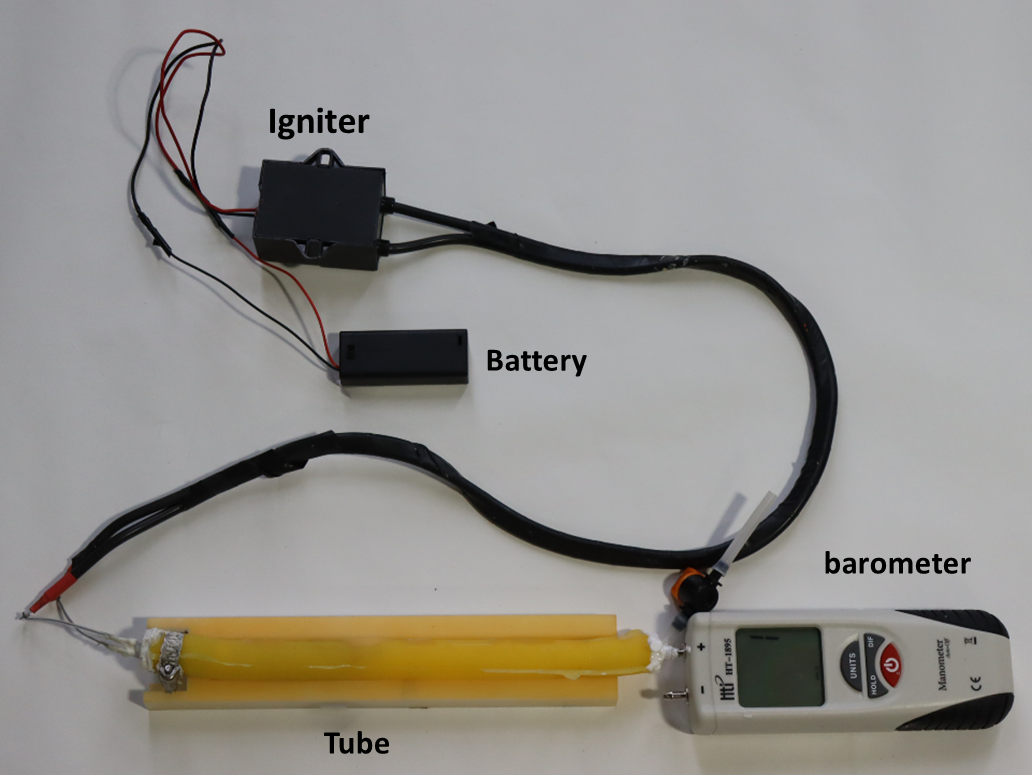
**

**Figure. S25.** The pressure test experimental setup inside the silicone tube.

Figure. S26.


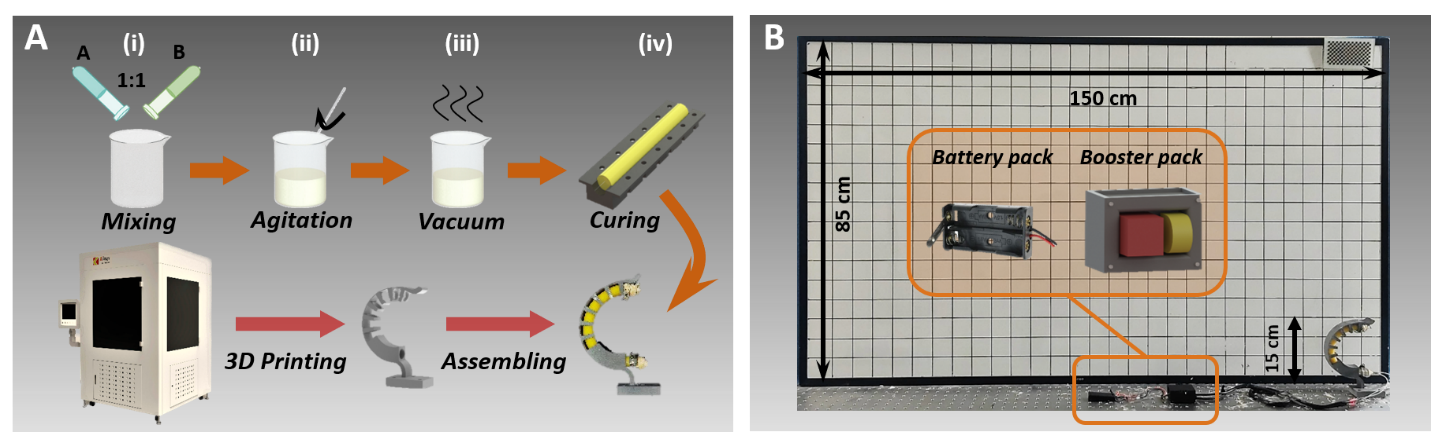


**Figure. S26.** (A) The manufacturing process of the actuator. (B) The experimental setup of the actuator actuating tests.

Figure. S27.

**
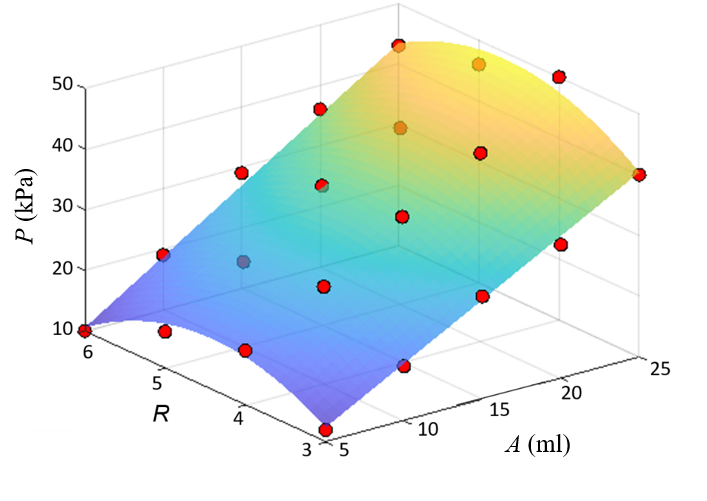
**

**Figure. 27.** The surface fitting of the pressure during the combustion.

Figure. S28.


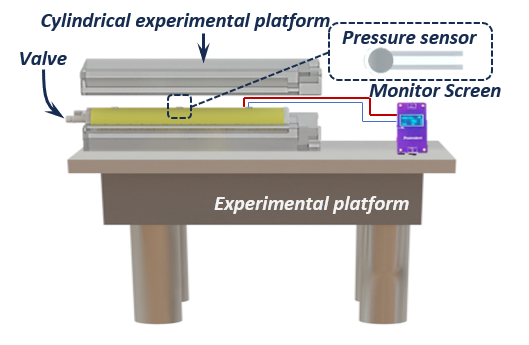


**Figure. S28.** The pressure experiment of the combustion-driven soft actuator.

Figure. S29.


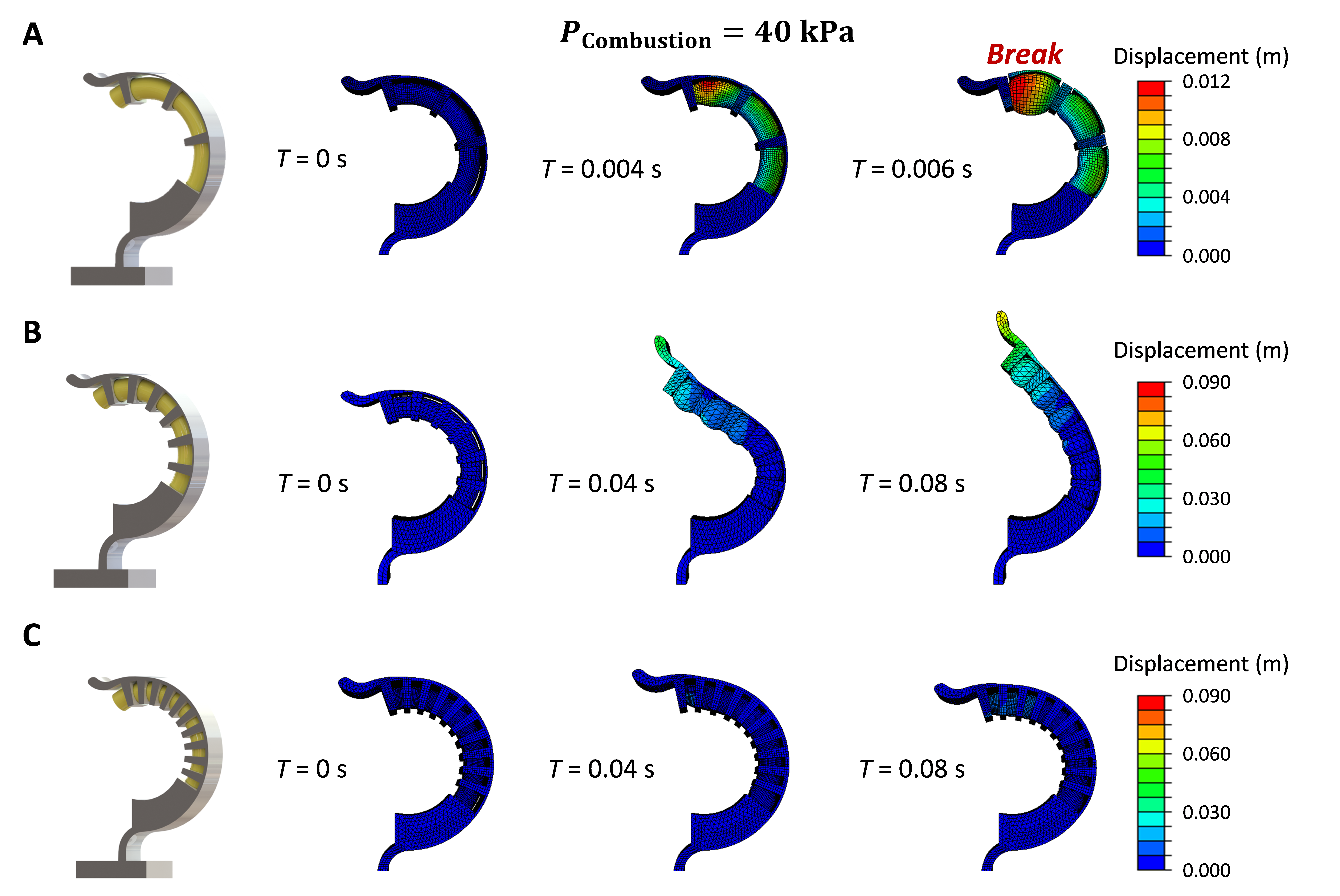


**Figure. S29.** The simulation of the combustion-driven soft actuator with different backbones and the $P_{\mathrm{Combustion}}=40 kPa$. (A) The simulation of the three backbones combustion-driven soft actuator, the soft actuator is damaged when the $T=0.006 s$. (B) The simulation of the six backbones combustion-driven soft actuator. (C) The simulation of the ten backbones combustion-driven soft actuator.

Figure. S30.


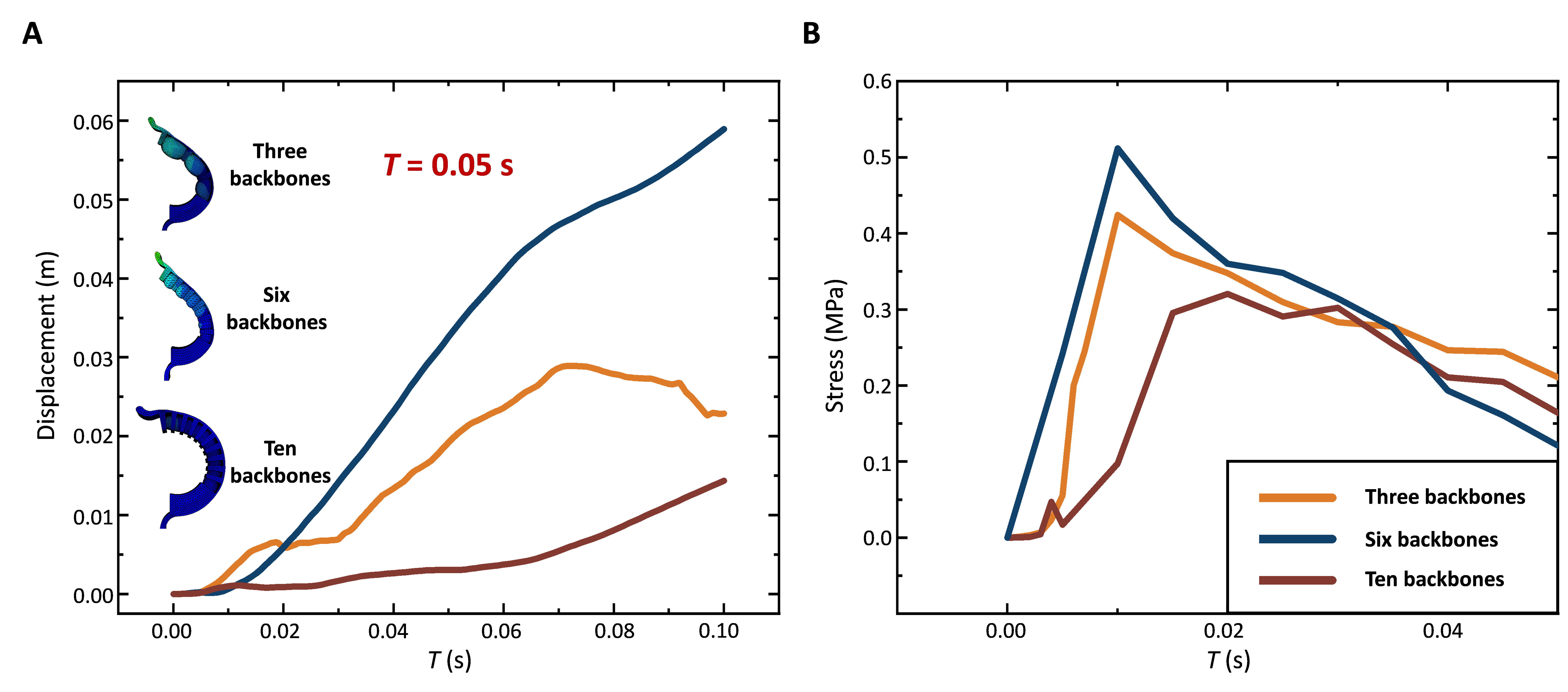


**Figure. S30.** The displacement and stress of combustion-driven soft actuator. (A) The top displacement of the combustion-driven soft actuator during actuation. (B) The top stress of the combustion-driven soft actuator during actuation.

Figure. S31.


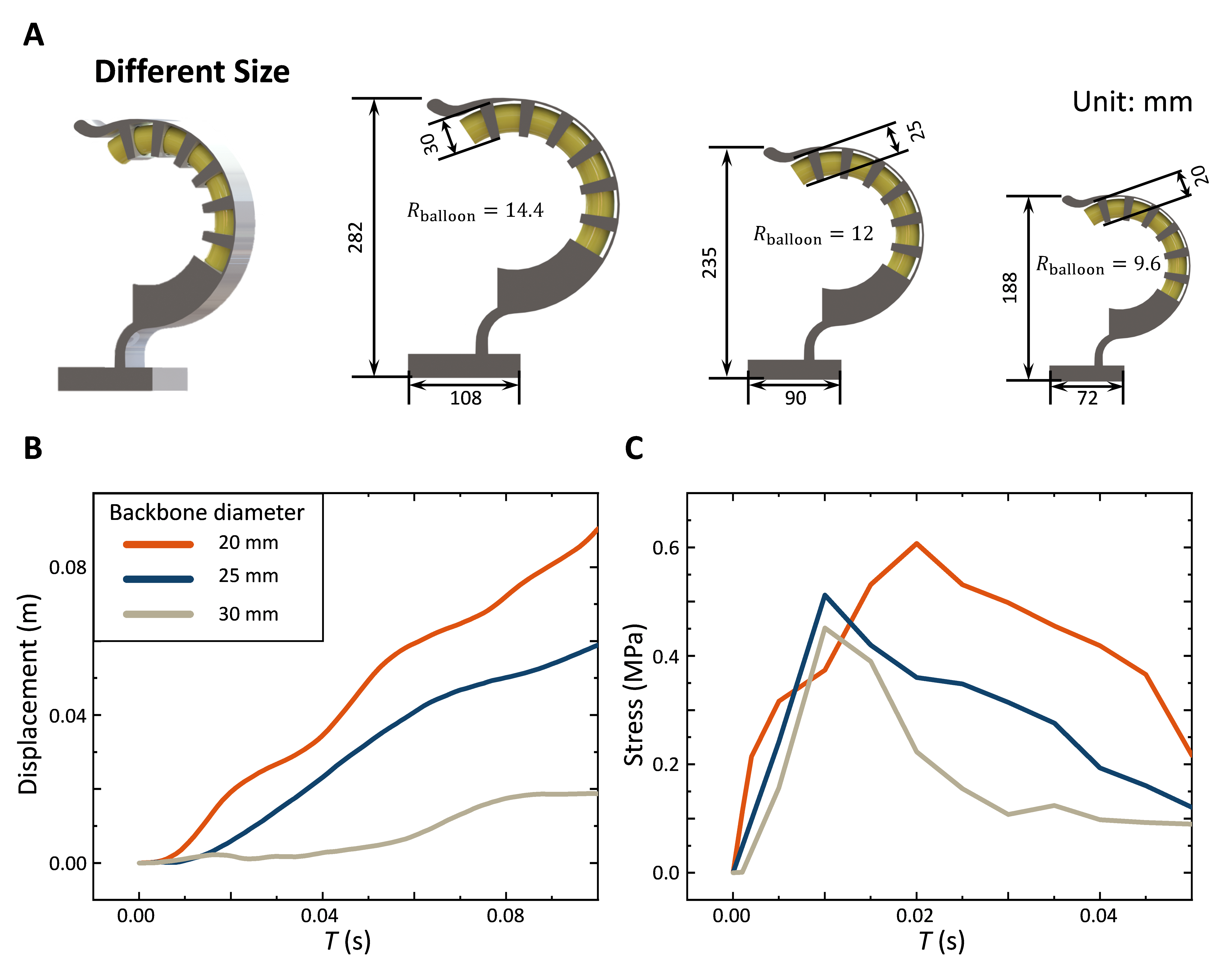


**Figure. S31.** The simulation of the combustion-driven soft actuator different geometric dimensions. (A) The design principle of the combustion-driven soft actuator with different geometric dimensions, backbone diameter is 30 mm, 25 mm, and 20 mm. (B) The top displacement of the combustion-driven soft actuator during actuation. (C) The top stress of the combustion-driven soft actuator during actuation.

Figure. S32.


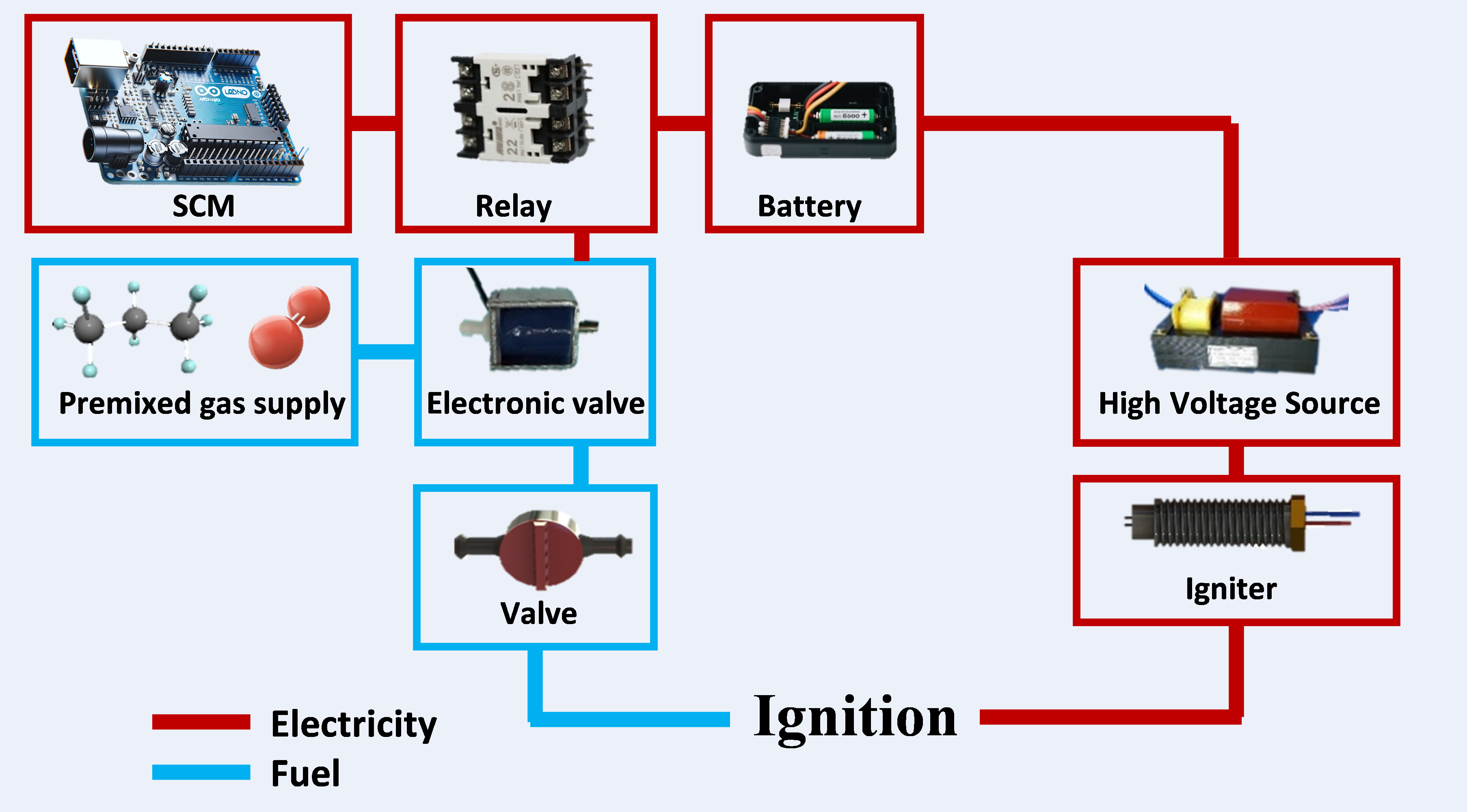


**Figure. S32.** Combustion-driven soft actuator automatic drive schematic diagram.

Figure. S33.


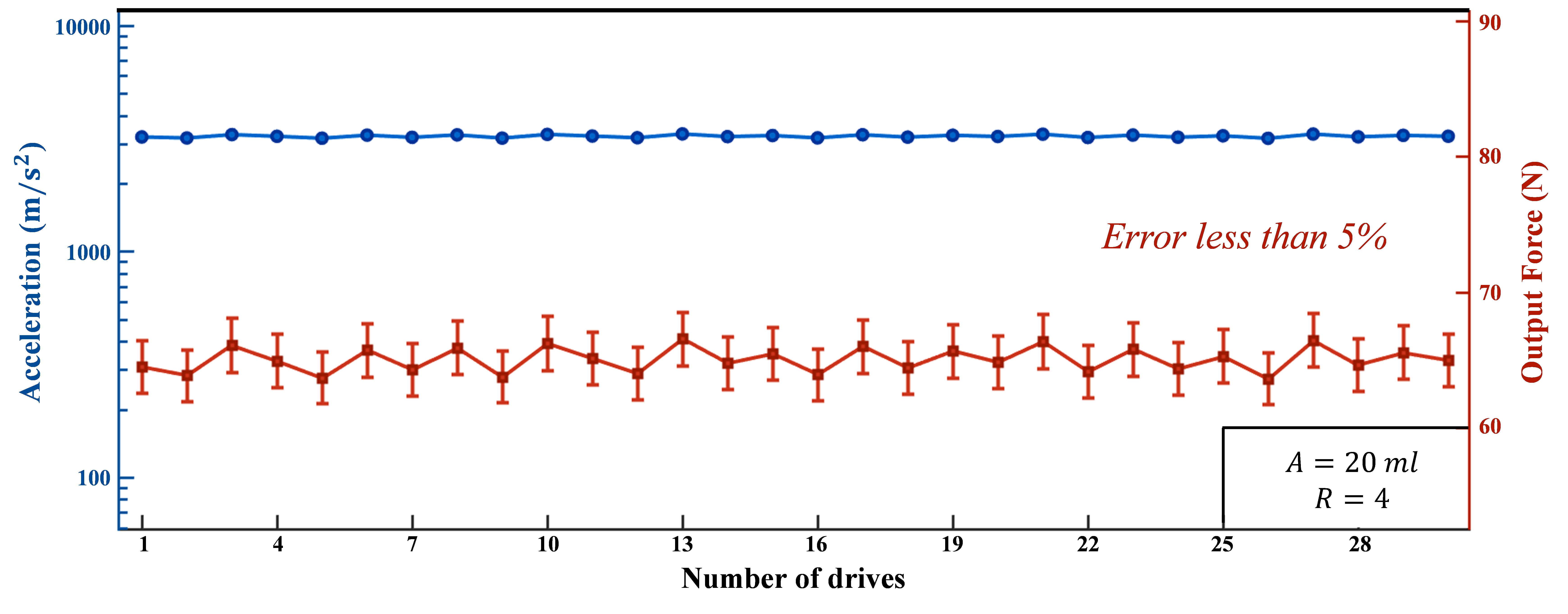


**Figure. S33.** Combustion-driven soft actuator performance over 30 cycles: By calculating the peak acceleration of the ball propelled by the actuator, the output force error is less than 5%. When air volume ($A$) is 20 ml and the ratio ($R$) is 4.

Table S1.

**Table S1. The details of simulation with different combustion stresses**

| Case | Combustion stresses (Pa) | Backbone material | Young's modulus (MPa) |
| --- | --- | --- | --- |
| 1 | 12500 | Nylon | 1646 |
| 2 | 18750 | Nylon | 1646 |
| 3 | 25000 | Nylon | 1646 |
| 4 | 31250 | Nylon | 1646 |
| 5 | 37500 | Nylon | 1646 |

Table S2.

**Table S2. The details of simulation with different backbone material**

| Case | Combustion stress (Pa) | Backbone material | Young's modulus (MPa) |
| --- | --- | --- | --- |
| 1 | 26250 | Nylon | 1646 |
| 2 | 26250 | Resin | 2400 |
| 3 | 26250 | PLA | 3500 |

Movie S1. The experimental video of combustion-enabled soft actuator’s actuating test when the ratio of oxygen and propane is 4:1 and the gas amount is 14 ml.

Movie S2. The three-point shooting demonstration of combustion-enabled soft actuator.

Movie S3. The baseball home run demo of combustion-enabled soft actuator which can hit powerfully.

Movie S4. The demonstration of the combustion-driven actuator horizontal sensing and driving system.

Movie S5. The combustion-driven Catbot jumping demonstration which can jump for a long distance with high speed.

Movie S6. The motion performance of the Jump-and-Fly Catbot.

Movie S7. Multiple flight motions of the Jump-and-Fly Catbot.

Movie S8. The applications of the Jump-and-Fly Catbot jumping from complex terrain.

Movie S9. The applications of the Jump-and-Fly Catbot rapid extrication from tree branches.

Movie S10. Rapid escape from catching net demonstration of three types robots, including Jump-and-Fly Catbot, a conventional UAV, and a jumping robot.
